# Open source scientific bottle roller

**DOI:** 10.1016/j.ohx.2023.e00445

**Published:** 2023-06-22

**Authors:** Maryam Mottaghi, Yuntian Bai, Apoorv Kulkarni, Joshua M. Pearce

**Affiliations:** a Department of Mechanical and Materials Engineering, Western University, 1151 Richmond St., London, Ontario N6A 5B9 Canada; b Department of Electrical and Computer Engineering, Western University, 1151 Richmond St. London, Ontario N6A 5B9 Canada; c Department of Electrical and Computer Engineering, Ivey Business School, Western University, 1151 Richmond St. London, Ontario N6A 5B9 Canada

**Keywords:** Bottle roller, 3D printing, Chemical mixing, Bottle rolling, Open hardware, Scientific hardware

## Abstract

Proprietary bottle rolling systems automate some laboratory applications, however, their high costs limit accessibility. This study provides designs of an open source bottle roller that is compatible with distributed digital manufacturing using 3-D printed parts and readily-available commercial components. The experimental results show that the open source bottle roller can be fabricated for CAD$210 (about USD$150) in materials, which is 86% less expensive than the most affordable proprietary bottle roller on the market. The design, however, is more robust with enhanced capabilities. The design can be adapted to the user’s needs, but is already compatible with incubators with a low profile (dimensions 50 cm x46 cm x8.8 cm) and capable of being operated at elevated temperatures. The systems can be adjusted to revolves from 1 to 200 RPM, exceeding the rotational speed of most commercial systems. The open source bottle roller as tested has a capacity greater than 1.2 kg and can roll twelve 100 mL bottles simultaneously. Validation testing showed that it can operate for days at 80 RPM without human intervention or monitoring for days at both room temperature and elevated temperatures (50 °C). Future work includes adapting the designs for different sizes and for different fabrication techniques to further reduce costs and increase flexibility.

Specifications table.

**Table:** 

Hardware name	*Open Source Scientific Bottle Roller*
Subject area	• Engineering and materials science • Chemistry and biochemistry
Hardware type	• Mechanical engineering and materials science
Closest commercial analog	*Thermo Scientific Bottle/Tube Roller, US$1276.89 (CAD$*1,539) (https://www.tequipment.net/Thermo-Scientific/Bottle/Tube-Roller/Tube-Roller-Mixers)
Open source license	*Documentation:* GNU General Public License (GPL) 3.0;Hardware: CERN OHL-S v2
Cost of hardware	*<USD$160 (CAD$210)*
Source file repository	https://osf.io/ps57u/
OSHWA certification UID	*CA000027*
DOI	https://doi.org/10.17605/OSF.IO/5ZRT3

## Hardware in context

A scientific bottle roller is used to rotate bottles at a set speed ranging from 1 revolution per minute (RPM) up to 80 RPM [1]. Bottle rollers are used in a range of scientific research areas such as cell cultivation [2], [3], sediment leaching [5], gold particulate separation [6], chemical blending [7], drying [8], and many other applications outside of the lab such as mixing essential oils or bituminous mixtures [9]. Although commercial bottle rolling systems automate some of these laboratory applications, their costs limit accessibility as the retail price of a simple single-layer bottle roller costs CAD$1540 (US$1136.97) [10] and those that are used in incubators can cost over CAD$2910 (US$2148.44) [11]. The large costs of proprietary scientific bottle rollers summarized in Table 1 limit accessibility in the scientific community. It should be noted that the CAD$ to US$ exchange rate used was about 1.355.

**Table 1:** Proprietary commercial bottle roller costs and specifications.

**Commercial Proprietary Product**	**Cost**	**Specifications**
Thermo Scientific Bottle/Tube Roller [10]	CAD$1,539.00 (US$1136.24)	• Speed Range: 1 to 80 RPM • Temperature Range: 4 °C to 60 °C • Number of rollers: 6
Fisherbrand Digital Bottle Roller [39]	CAD$1,888.98 (US$1394.62)	• Speed Range: 1 to 80 RPM • Temperature Range: 4 °C to 60 °C • Number of rollers: 3
Bottle/Tube Roller [1]	CAD$2,188.11 (US$1615.47)	• Speed Range: 2 to 38 RPM • Temperature Range: 4 °C to 60 °C • Number of rollers: 2
Scientific Low-Profile Bottle Roller [40]	CAD$3,239.98 (US$2392.06)	• Speed Range: 1 to 80 RPM • Temperature Range: 0 °C to 60 °C • Number of rollers: 10
FlexiRoll Digital Tube/Bottle Roller Shaker [41]	CAD$3,329.86 (US$2458.42)	• Speed Range: 0.5 to 80 RPM • Temperature Range: 4 °C to 60 °C • Number of rollers: 15–20

One approach to reducing research equipment costs is to use decentralized production of free and open source hardware (FOSH) [12], [13]. The FOSH approach can reduce costs [14], [15], enable customization and increase control for scientists [13], [15], [16], [17]. This is largely due to the development of open source digital manufacturing technologies such as the self-replicating rapid prototyper (RepRap) 3-D printer [18], [19], [20]. In this open hardware model [21], [22] designs are shared for free and then transformed to physical products through digital manufacturing. A review of the recent FOSH literature found scientific FOSH material costs had an average savings of 87% compared to equivalent or lesser proprietary tools [17]. These economic savings increased slightly to 89% for those that used open-source electronics like Arduino technology [23], 92% for those that implemented RepRap-class 3-D printing, and 94% with both [17]. When scientists build their own hardware [22], [24] using parametric FOSH [21], [25], it allows for high-quality bespoke research equipment [12], [26], [27], [28].

The FOSH approach has been applied to chemical mixing in several ways such as: i) an open source 3-D printed nutating mixer [29], ii) rotator mixer and shaker [30], iii) orbital shaker [31], iv) stirring [32] and v) a shake table [33]. Open source chemical mixing has also been used at the micro-scale for microfluidic devices [34] as well as for reactionware [35] using a wide variety of readily available chemically compatible feedstocks [36]. In addition, open source alternatives are available for pharmaceutical applications [37], and incubation via the Incubot for long-term live cell imaging [38]. Bottle rolling, however, has not yet been open sourced using a design that can be readily manufactured using a digital distribution model.

This study aims to overcome this limitation by reducing the cost of bottle rollers using the open hardware approach and distributed digital manufacturing. Specifically, this study provides the designs for an open source bottle roller that is a less expensive alternative to commercial bottle rolling systems, while also increasing the capacity of the bottle roller to allow for fewer bottle rolling systems to be used to complete a larger task. The open source bottle roller is manufactured using 3-D printed parts for custom mechanical parts and readily-available components for the power supply, rollers, bearings, and speed controller.

## Hardware description

The open-source bottle roller can be printedon any thermoplastic materials extrusion-based 3-D printer, but to overcome commercial limitations, the main components of the device are fabricated using a RepRap-class fused filament 3-D printer. Furthermore, the electronic parts, rollers, bearings, and speed controller are provided from readily available components in the local markets. The open source bottle roller is fully customizable and allows the user to increase the capacity of bottles on a larger scale. Testing and validation are provided to compare the quality of the open source bottle roller with the available commercial ones, and a comparison in price is made to show the economic advantage of the open-source one with commercial peers. The features of the open source bottle roller include:

• Low-cost chemical mixing for laboratory purposes
• Customizable design based on the user’s needs
• Compatible with incubators with a low profile (dimensions 50 cmx46 cmx8.8 cm)
• Revolves 1 to 200 RPM, while the maximum speed of most commercial systems is 80 RPM
• Operational in an incubator at elevated temperatures (50 °C)
• Holds 12 bottles of 100 mL simultaneously. (Capacity greater than 1.2 kgs)

## Design files

### Design files summary

All files are at https://osf.io/ps57u/ and released under GNU General Public License (GPL) 3.0 for documentation and CERN OHL-S v2 for hardware. The DOI for the repository is https://doi.org/10.17605/OSF.IO/PS57U and the registration is https://doi.org/10.17605/OSF.IO/5ZRT3 The design file information is summarized in Table 2.

**Table 2:** Design file information.

Component name and rendered image	File type	OS License	Quantity needed to be printed	Location of the file
Design file 1 (Axis)	STL, STEP, CAD	CERN OHL-S v2	5	https://osf.io/u7ykc https://osf.io/uyk5z https://osf.io/kxh3b
Design file 2 (Belt)	STL, STEP, CAD	CERN OHL-S v2	5	https://osf.io/gkupa https://osf.io/rxzuy https://osf.io/h74b5
Design file 3 (Gear-1)	STL, STEP, CAD	CERN OHL-S v2	2	https://osf.io/mcn9e https://osf.io/5dxg7 https://osf.io/2q8j6
Design file 4 (Gear-2)	STL, STEP, CAD	CERN OHL-S v2	3	https://osf.io/hf95r https://osf.io/wg7p6 https://osf.io/7htce
Design file 5 (Motor Gear)	STL, STEP, CAD	CERN OHL-S v2	1	https://osf.io/pwc8m https://osf.io/by8jr https://osf.io/xyc3k
Design File 6 (Half Connector)	STL, STEP, CAD	CERN OHL-S v2	4	https://osf.io/jzkga https://osf.io/kh3sy https://osf.io/8u57d
Design file 7 (Wall-1)	STL, STEP, CAD	CERN OHL-S v2	1	https://osf.io/7ngzh https://osf.io/w58fa https://osf.io/6utmw
Design file 8 (Wall-2)	STL, STEP, CAD	CERN OHL-S v2	1	https://osf.io/6cx3u https://osf.io/tdav4 https://osf.io/jnph5
Design file 9 (Wall-3)	STL, STEP, CAD	CERN OHL-S v2	1	https://osf.io/c5td7 https://osf.io/dthjq https://osf.io/mcnq6
Design file 10 (Wall-4)	STL, STEP, CAD	CERN OHL-S v2	1	https://osf.io/epj7z https://osf.io/rba36 https://osf.io/7ykte
Design file 11 (Outer Plate-1)	STL, STEP, CAD	CERN OHL-S v2	1	https://osf.io/4j6ys https://osf.io/ahmky https://osf.io/96z7y
Design file 12 (Outer Plate-2)	STL, STEP, CAD	CERN OHL-S v2	1	https://osf.io/md59j https://osf.io/vc8ax https://osf.io/uf2qz
Design file 13 (Outer Plate-3)	STL, STEP, CAD	CERN OHL-S v2	1	https://osf.io/njsrv https://osf.io/spz8g https://osf.io/hc2vn
Design file 14 (Outer Plate-4)	STL, STEP, CAD	CERN OHL-S v2	1	https://osf.io/3kabt https://osf.io/ktzsw https://osf.io/dmrtx
Design file 15 (Reinforce Bar)	STL, STEP, CAD	CERN OHL-S v2	4	https://osf.io/k8n47 https://osf.io/x7vnp https://osf.io/skchx
Design file 16 (Roller Mount)	STL, STEP, CAD	CERN OHL-S v2	10	https://osf.io/37sr2 https://osf.io/feax3 https://osf.io/zt3ng
Design file 17 (Spacer)	STL, STEP, CAD	CERN OHL-S v2	10	https://osf.io/e429f https://osf.io/es69f https://osf.io/zh275
Design file 18 (Motor Box)	STL, STEP, CAD	CERN OHL-S v2	1	https://osf.io/xe5kw https://osf.io/khqnx https://osf.io/g7u94
Design file 19 (Cover-1)	STL, STEP, CAD	CERN OHL-S v2	1	https://osf.io/qv7zf https://osf.io/m9b4e https://osf.io/wxj5u
Design file 20 (Cover-2)	STL, STEP, CAD	CERN OHL-S v2	1	https://osf.io/73e9j https://osf.io/cf2kx https://osf.io/qaj9v
Design file 21 (Wire Cover-1)	STL, STEP, CAD	CERN OHL-S v2	1	https://osf.io/brmw8 https://osf.io/hdeyk https://osf.io/y7nxz
Design file 22 (Wire Cover-2)	STL, STEP, CAD	CERN OHL-S v2	1	https://osf.io/m3dku https://osf.io/ydkfb https://osf.io/2p6yu

**Table 3:** The bill of material list of hardware components to be purchased.

**Component**	**Number**	**Cost per unit -currency**	**Total cost - currency**	**Source of materials**	**Material type**
PETG filament	∼990 g	CAD$23.95 (per kg)	CAD$23.71	3D Printing Canada [43]	PETG
TPU filament	∼32 g	CAD$44.95 (per kg)	CAD$1.44	3D Printing Canada [44]	TPU
12v power supply	1	CAD$14.99	CAD$14.99	Amazon [45]	Power supply
12 V 1100 RPM DC motor	1	CAD$40	CAD$40	Alibaba [46]	DC motor
PWM speed controller	1	CAD$24.22	CAD$24.22	Amazon [47]	PWM controller
M5*30 mm bolts and nuts	2 sets	CAD$16.99	CAD$33.98	Amazon [48]	Screw set
1–1/2 in × 10 ft PVC Pipe	1	CAD$34.99	CAD$34.99	Lowes [49]	PVC
PGN 6005-2RS Sealed Ball Bearing − 25x47x12	10	CAD$2.7	CAD$27	Amazon [50]	Chrome Steel
Deep Groove Ball Bearing	2	CAD$8.99	CAD$8.99	Amazon [51]	Alloy Steel
			CAD$209.23		

*Tooling cost is not included. Tools including handsaw, wrench, screwdrivers, and soldering devices are needed during the assembly.

**Fig. 1:**
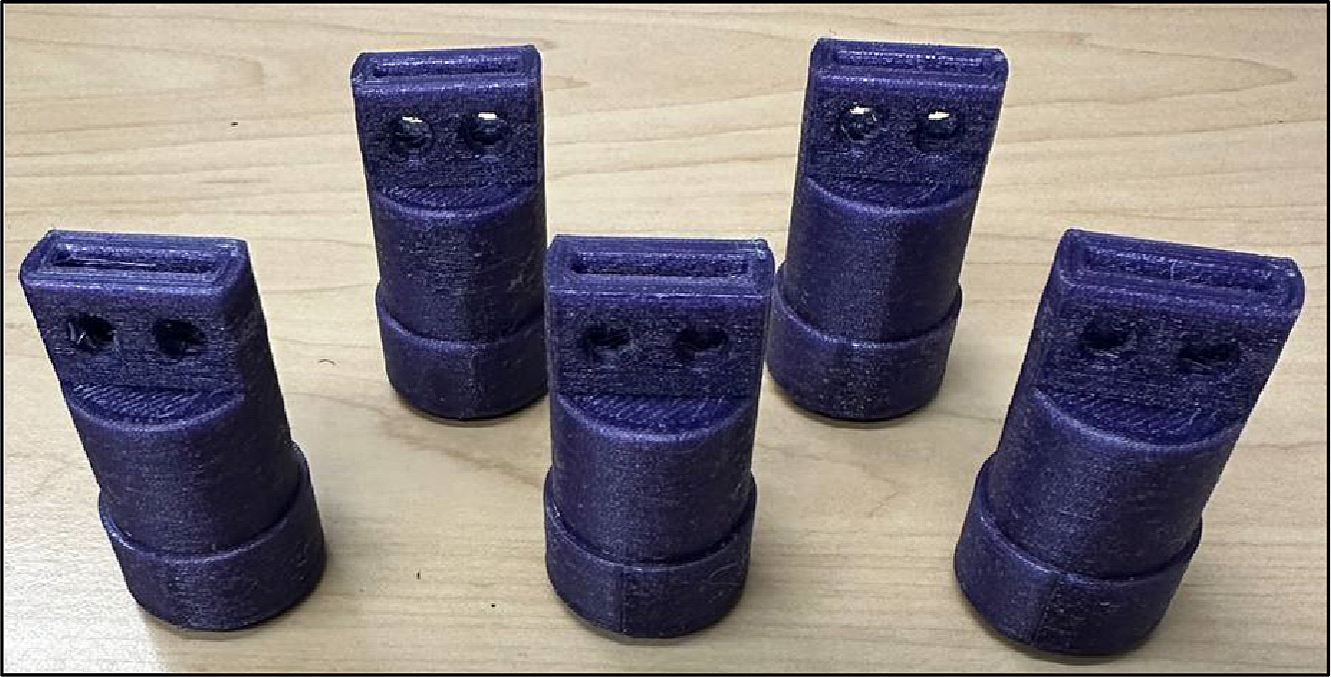
Axis.

File 1 represents the axis. This part connects the gear to the pipe (Fig. 1).

**Fig. 2:**
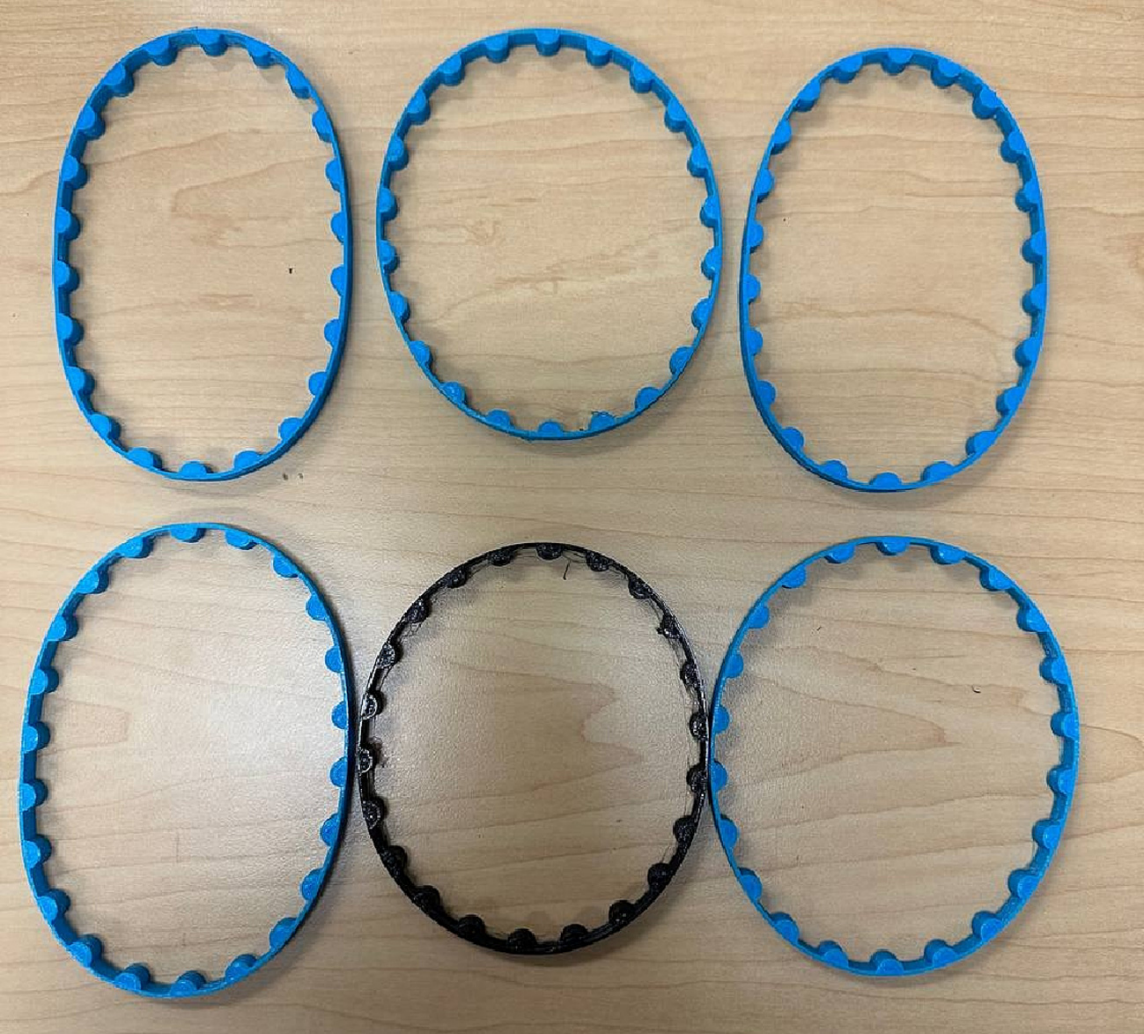
Belts.

In design file 2, the belts are shown. These belts connect the gears together to transfer the motor rotation to the pipes (Fig. 2). These belts and the gears are designed and customized based on the device’s size and replacing them with the readily available belts in the market is not recommended. Moreover, the quality of the belts can be changed by adjusting the infill percentage in the slicer settings. It should ne noted that the tension on the belts can be adjusted to the users wishes as the belts themselves can be customized and 3-D printed using flexible filament like NinjaFlex or similar.

**Fig. 3:**
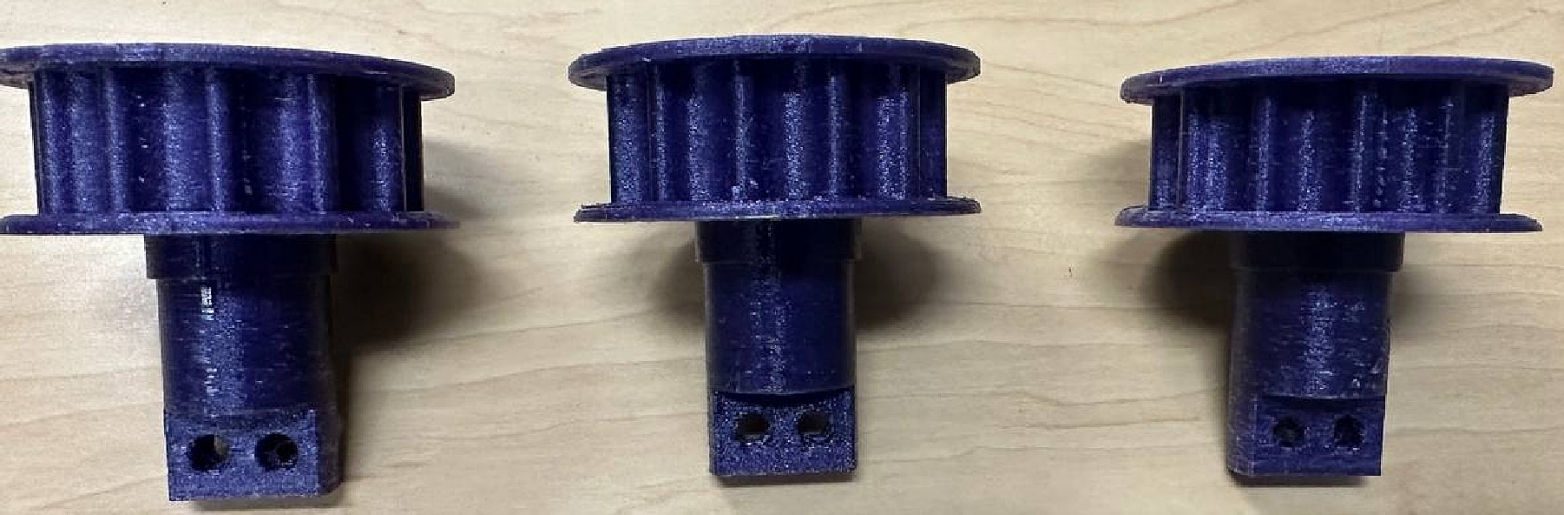
Gear-1.

In design file 3 gear 1 is shown. The gear is designed based on standard relationship between Pitch Diameter, center Distance, and belt Pitch Length [42] (Fig. 3).

**Fig. 4:**
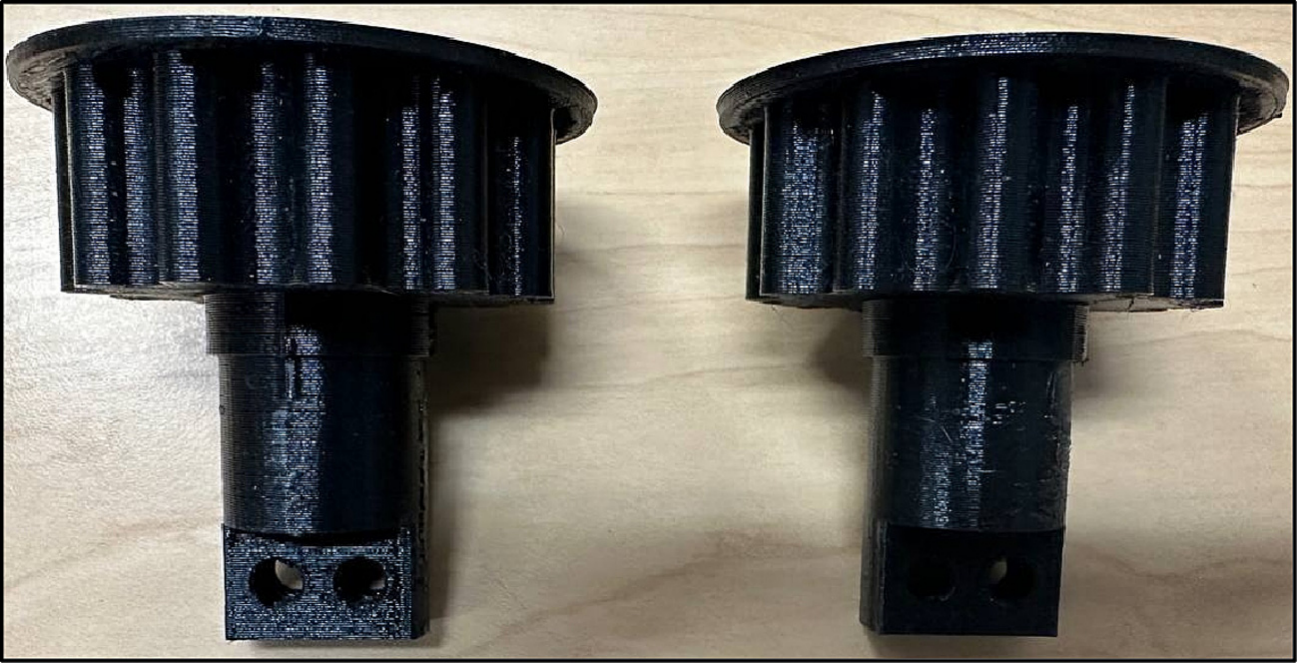
Gear-2.

The gear 2 is shown in design file 4. The gears are designed differently to easily accommodate the belts and reduce friction between them (Fig. 4).

**Fig. 5:**
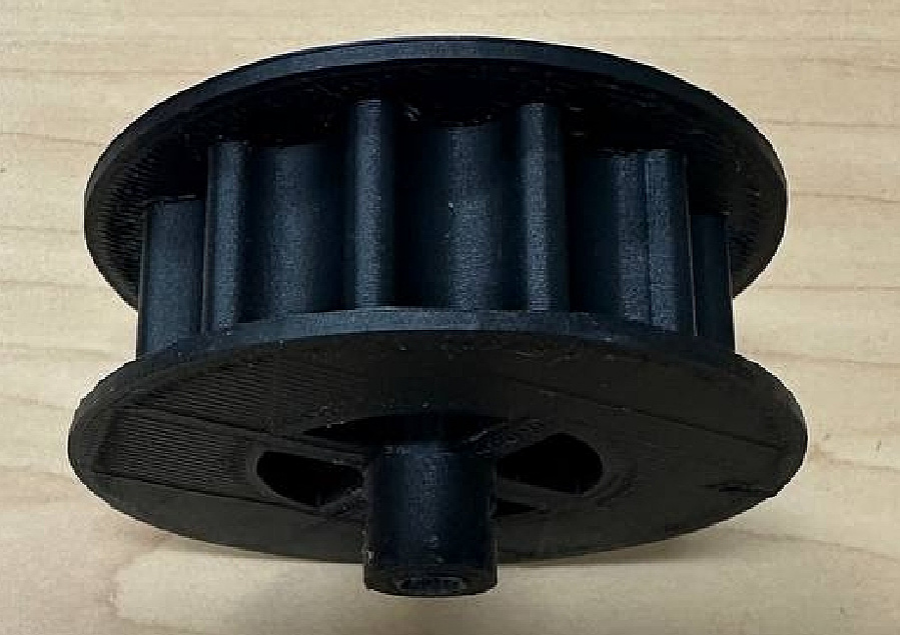
Motor Gear.

The shaft goes inside the horizontal hole, and it is held in place by inserting two screws in the vertical holes (Fig. 5).

**Fig. 6:**
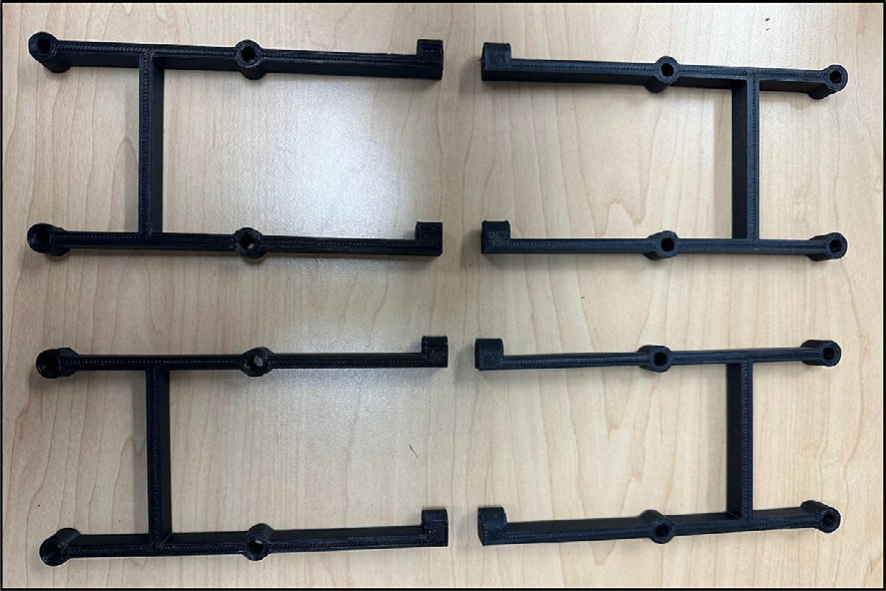
Half connector.

Since the main body is separated into 8 plates and walls in order to fit the print beds of most desktop 3-D printers, some connectors are needed to connect the plates together. Design file 6 represents these connectors (Fig. 6).

**Fig. 7:**
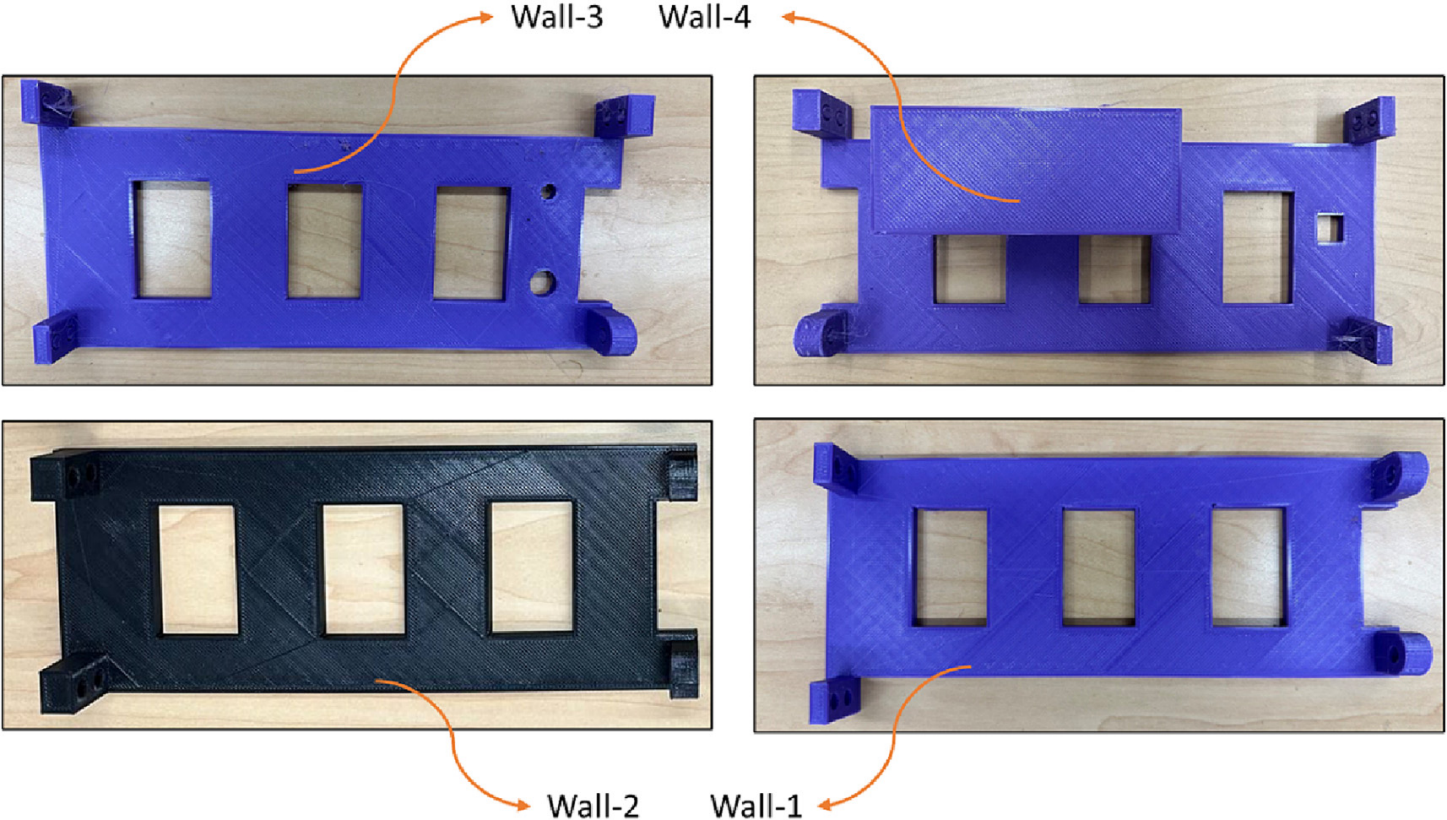
Wall-1, Wall-2, Wall-3, and Wall-4.

The walls that are parallel to the PVC pipes are shown in Fig. 7.

**Fig. 8:**
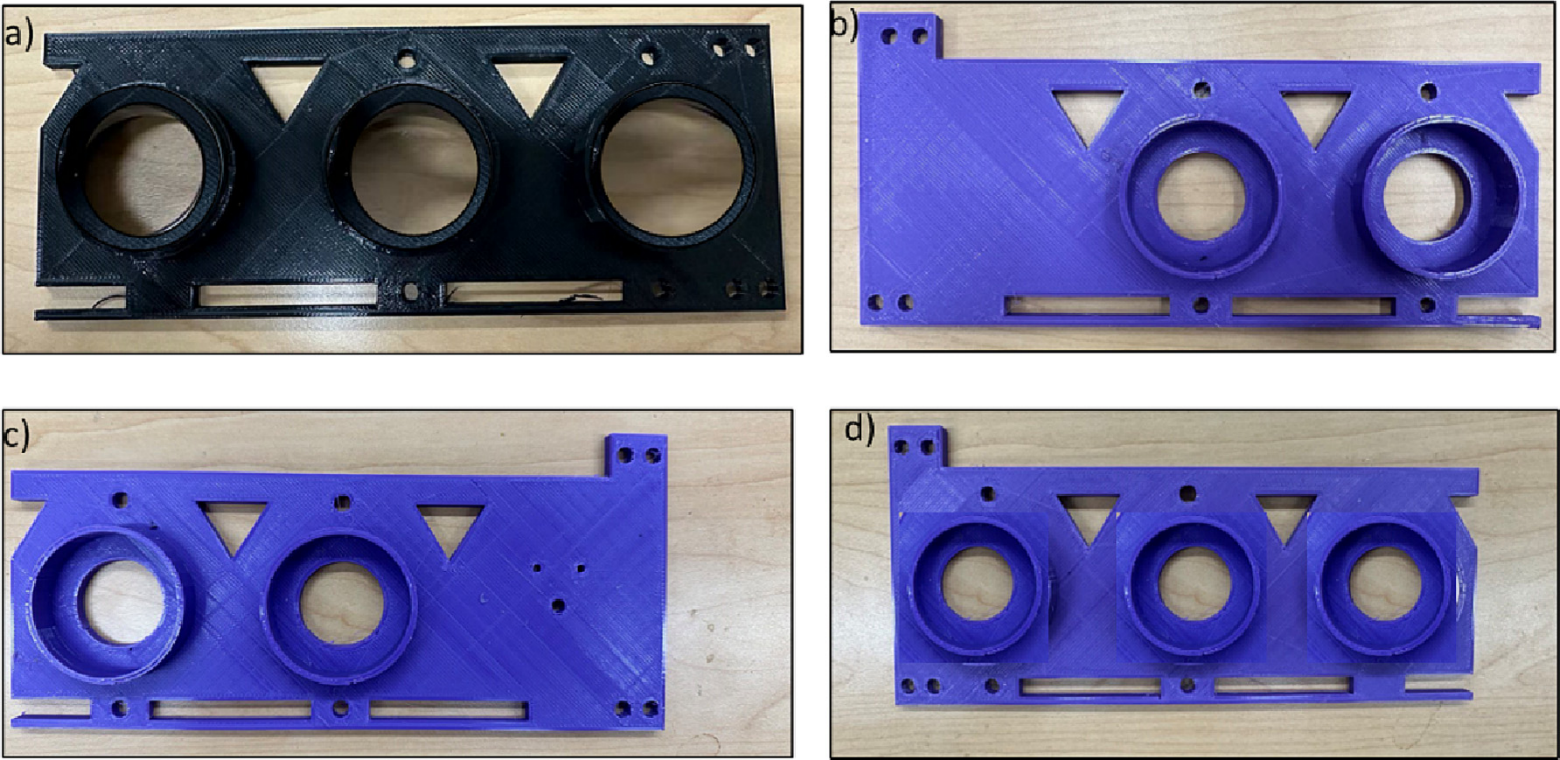
Outer Plates 1) Outer Plate-1, b) Outer Plate-2, c) Outer Plate-3, d) Outer Plate-4.

The outer plates are meant to connect the gears to the pipes. The holes are designed based on the diameter of the bearings (Fig. 8).

**Fig. 9:**
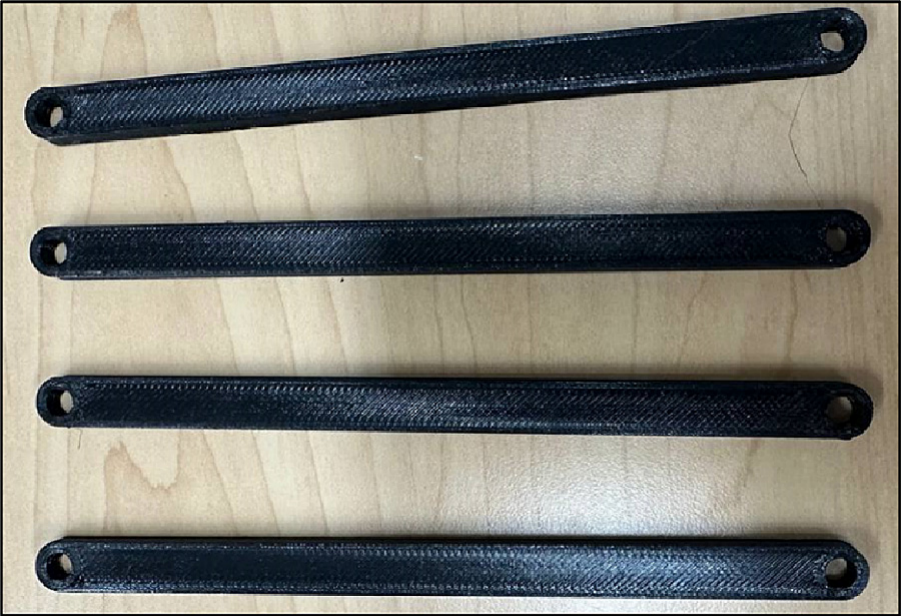
Reinforcing Bar.

The reinforcing bars that are shown in Fig. 9 are designed to keeps the main body from shaking while in operation (Fig. 9).

**Fig. 10:**
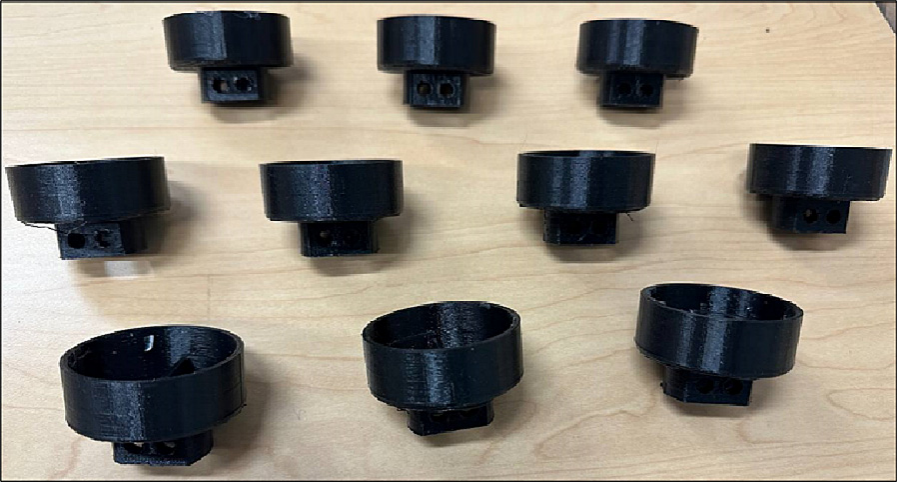
Roller Mount.

Design file 16 represents the roller mount that should be glued to the PVC pipes. The pipes are connected to the axis and then the gears by theses roller mounts (Fig. 10).

**Fig. 11:**
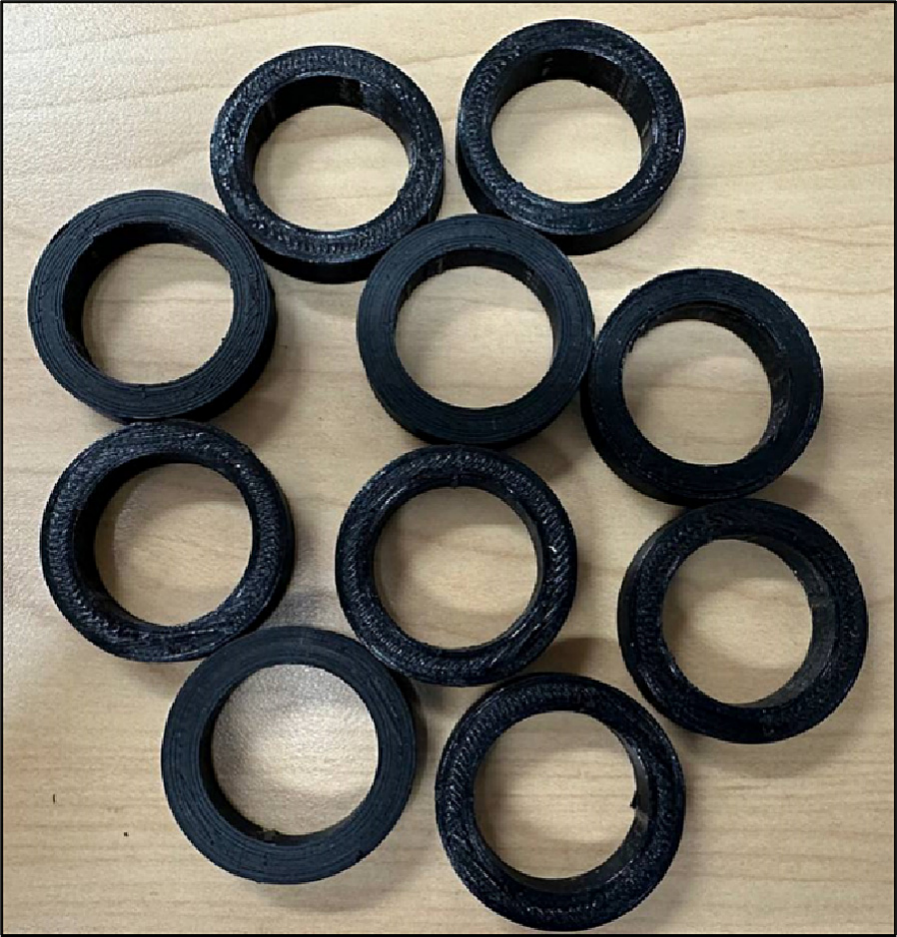
Spacer.

The spacer parts that are shown in Fig. 11 should be located between the roller mount and the axis to enable a proper distance between the roller mount and the bearings.

**Fig. 12:**
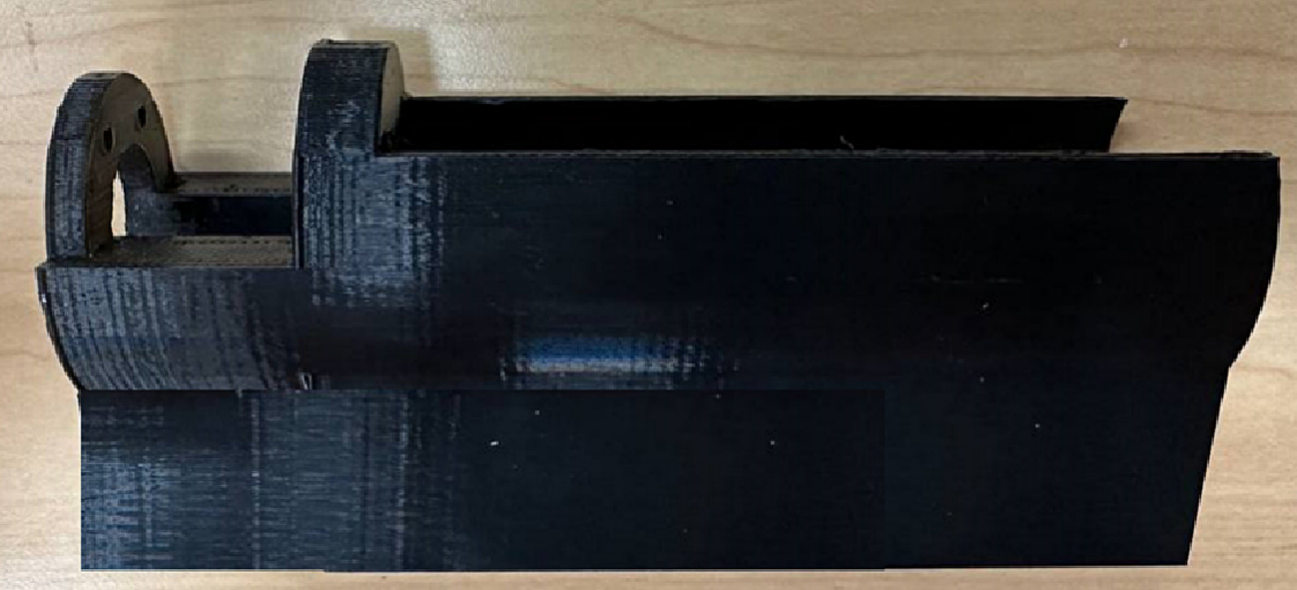
Motor box.

The motor box keeps the motor and the coupler held in place Fig. 12.

**Fig. 13:**
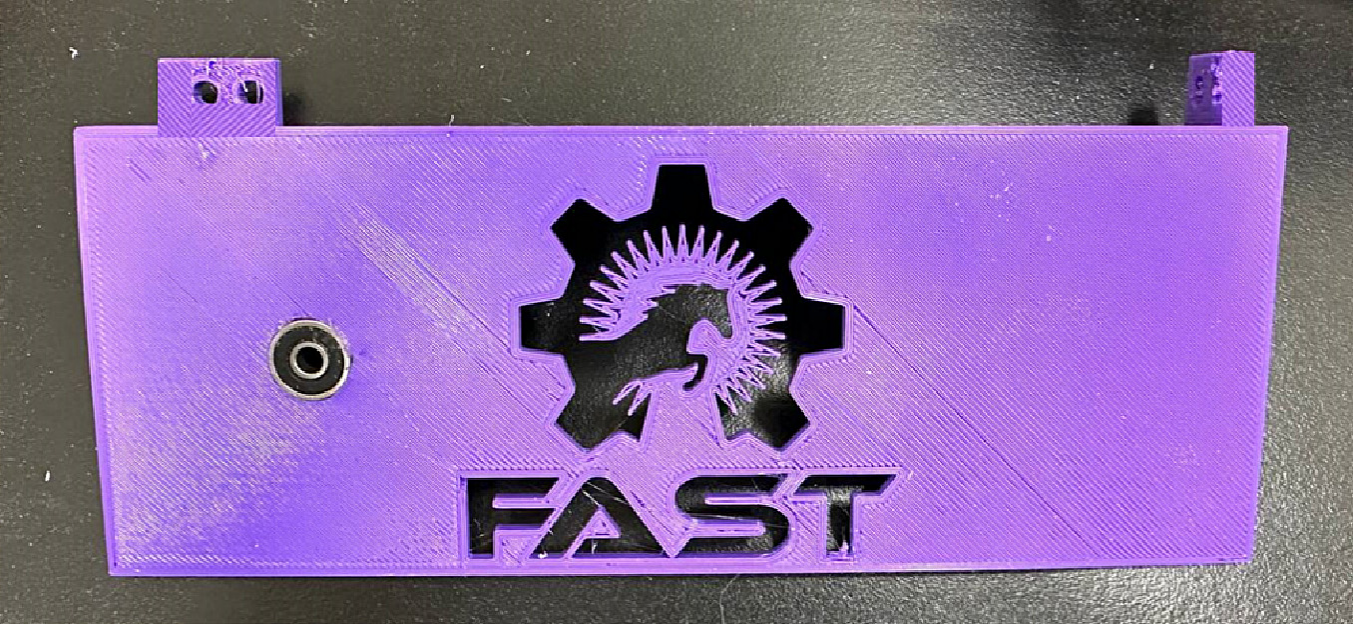
Cover-1.

**Fig. 14:**
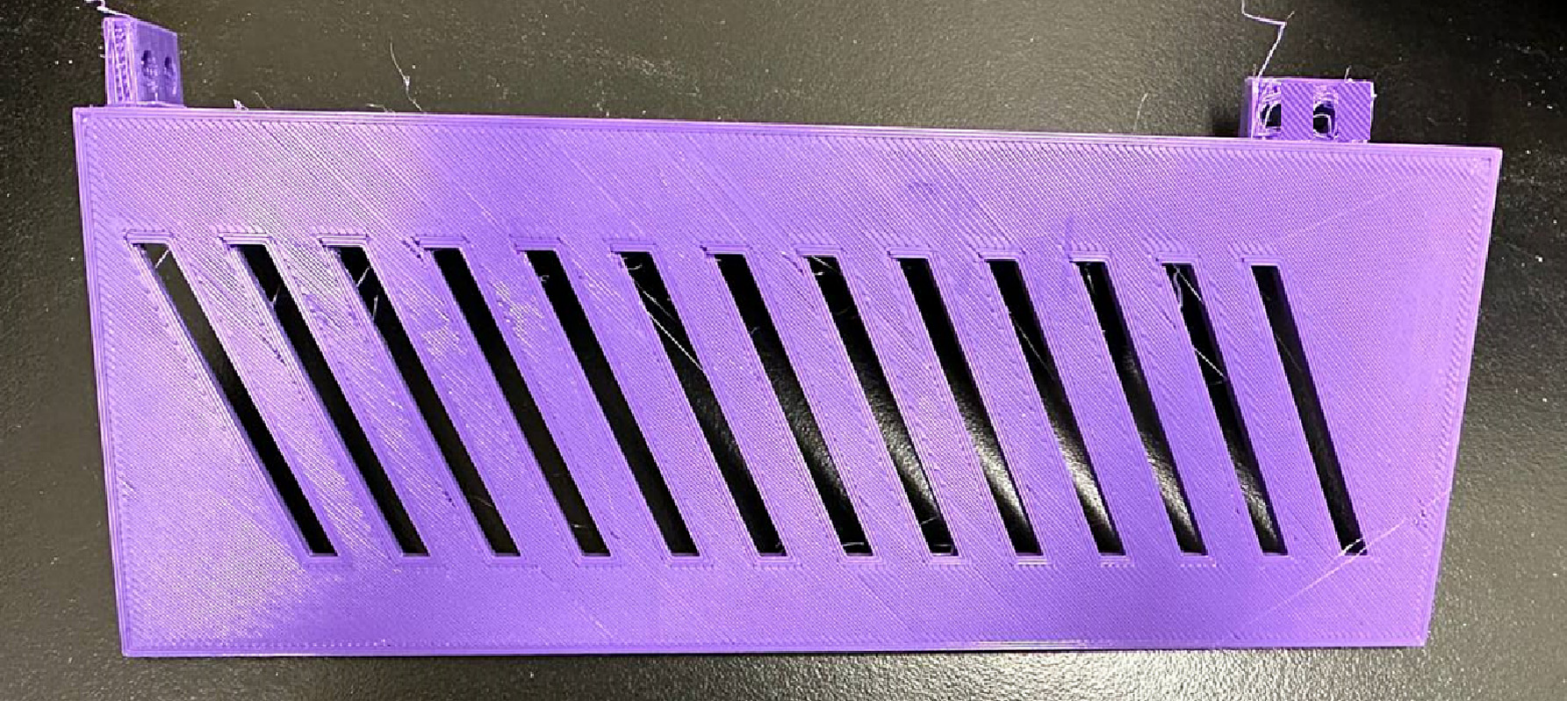
Cover-2.

The cover parts in file 19 and file 20 are designed to cover the gears and belts in order to prevent the operator's fingers from getting pinched in them. The printed parts are shown in (Fig. 13 and Fig. 14).

**Fig. 15:**
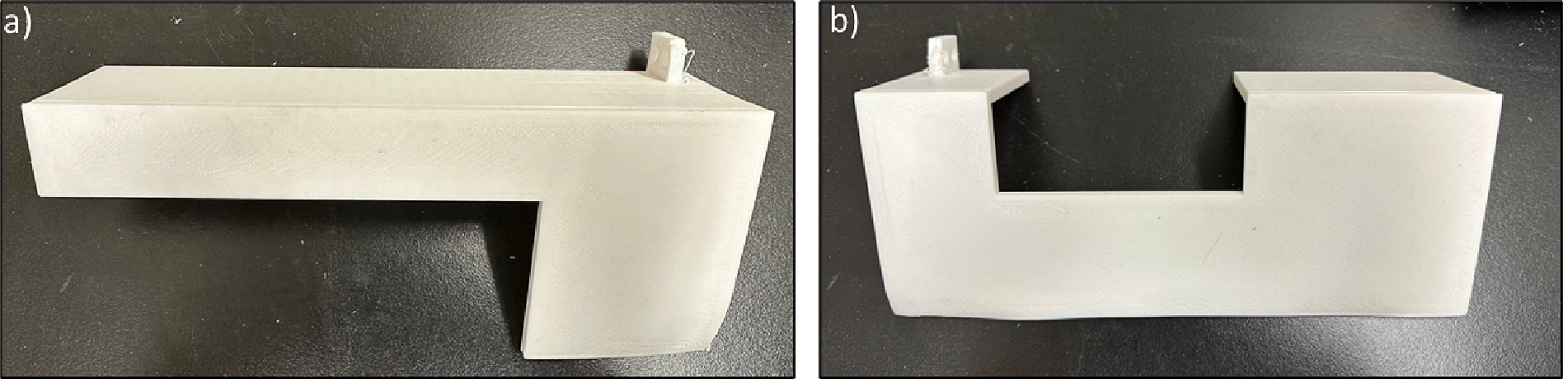
Wire cover-1 and Wire cover-2.

File 21 and File 22 represent the wire covers. Since there is a possibility of liquid spills from the bottles, these covers are designed to prevent the short circuit and reduce safety issues (Fig. 15).

**Fig. 16:**
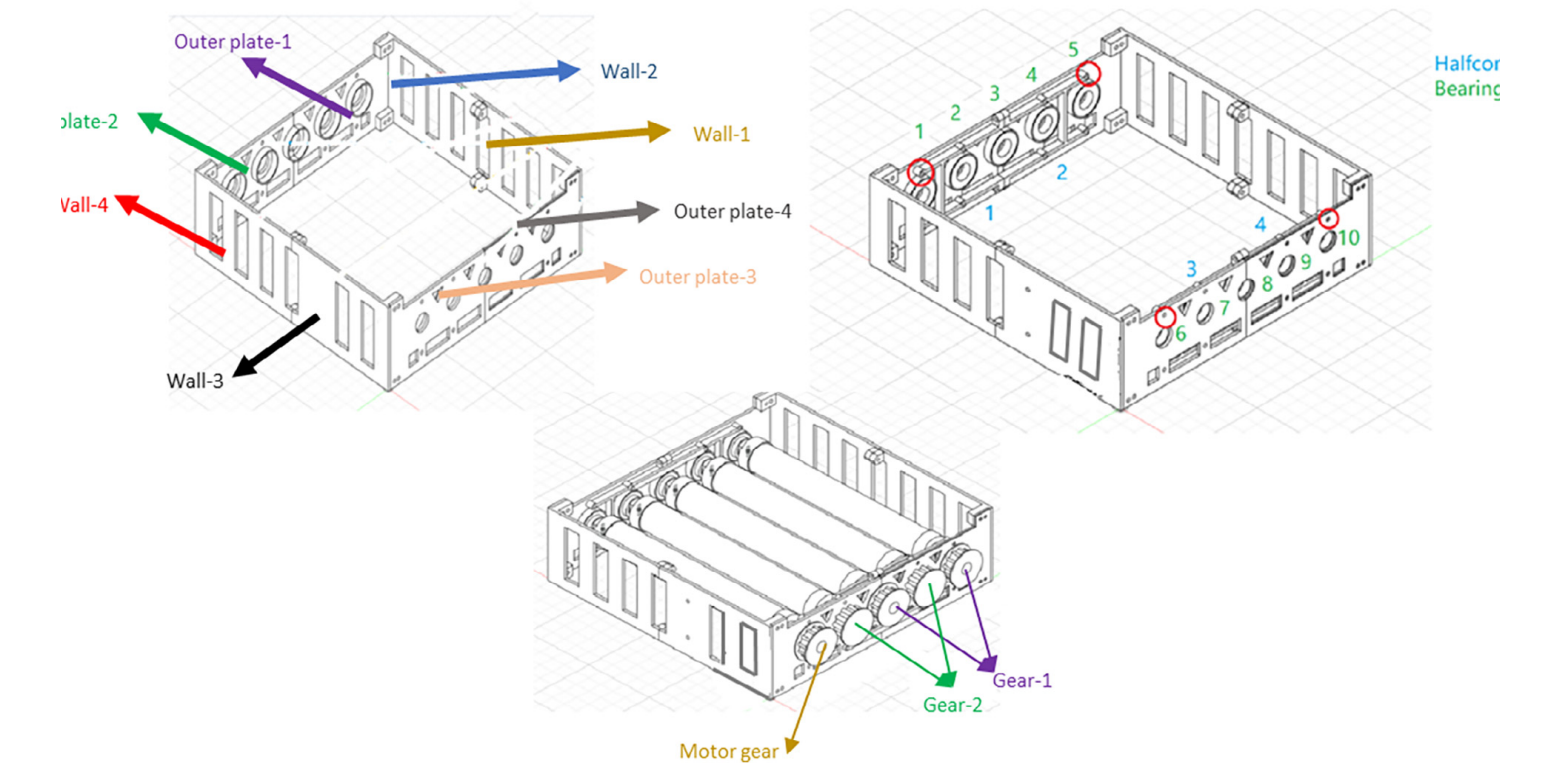
Frame part names.

The CAD file for the assembly of the bottle roller parts is represented by Design File 23. For further clarification, Fig. 16 displays the names of the different parts.

## Bill of materials

### Bill of materials summary

The overall design is based on the principle of maximizing strength and service life; thus, all components are designed to have maximum redundancy. Belts were selected as the primary transmission method because chains can cause additional wear and tear on the gears, whereas belts can be easily replaced as they are 3-D printable.. In a similar way, the electrical components are selected to be as simple as possible to ensure reliability, so a DC motor and PWM controller are chosen as the power source and speed controller. In addition, the design heavily relies on screws to achieve maximum link strength between printed components. In the aspect of performance, the finished prototype has five rollers to ensure enough room twelve 100 mL bottles to be mixed simultaneously. The motor allows the system to run at the range from 1 RPM to 200 RPM, and the power percentage of the motor can be monitored through the screen on the PWM controller.

### Build instructions

The first step for building the open source bottle roller is to 3-D print the parts listed in Table 2 using the STL files, which can be found on the OSF repository. The printing parameters are summarized in Table 4.. The main parts and belts were 3-D printed using PETG and TPU filaments, respectively. PETG was selected for the main parts due to their required strength, while TPU was chosen for the belts for its flexibility. Although the parts can be printed with any compatible 3-D printer, we used the open-source Lulzbot Taz 6 RepRap-class 3-D printer to produce the main parts, and the Lulzbot Sidekick 289 RepRap-class 3-D printer to print the belts, as this allowed for reduced costs.

**Table 4:** 3-D printing parameters.

**Parameter**	**Value**
Layer Height	0.18 mm
Initial Layer Height	0.425 mm
Wall Thickness	1 mm
Infill Density	20 %
Infill Line Distance	5 mm
Printing Temperature	210 ˚C
Build Plate Temperature	60 ˚C
Print Speed	60 mm/s
Infill Speed	40 mm/s
Wall Speed	30 mm/s
Travel Speed	175 mm/s
Initial layer Speed	15 mm/s
Support density	30 %

Next, the other parts from Table 3 must be acquired. The assembling steps are as follows:

1. Acquire all components.
2. Cut five sections of the PVC pipe according to Fig. 17. The cut does not need to be highly precise.

**Fig. 17:**
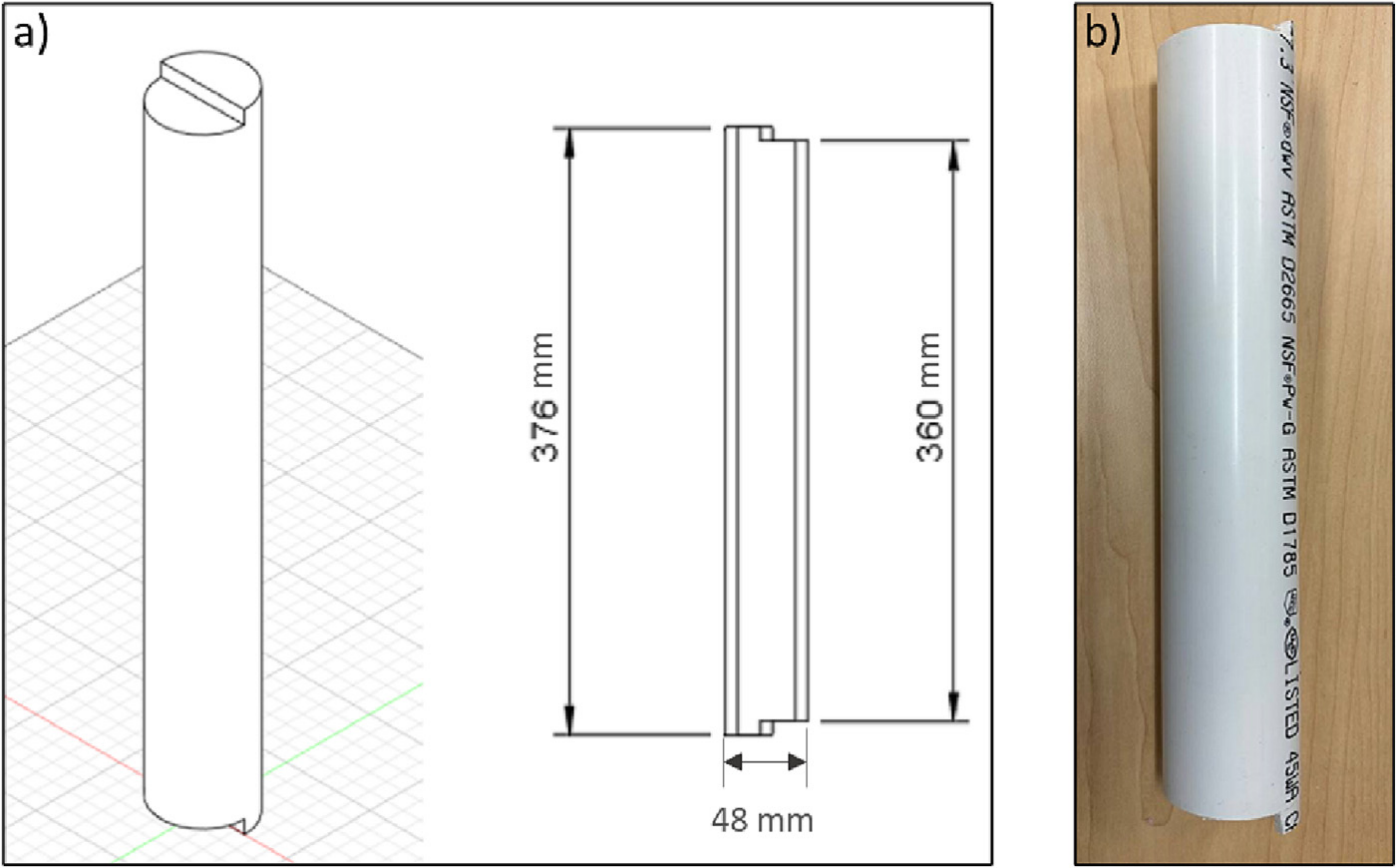
PVC pipe a) Drawing, b) Part.
3. Use epoxy glue to connect the Roller Mounts to the PVC pipes (Fig. 18).

**Fig. 18:**
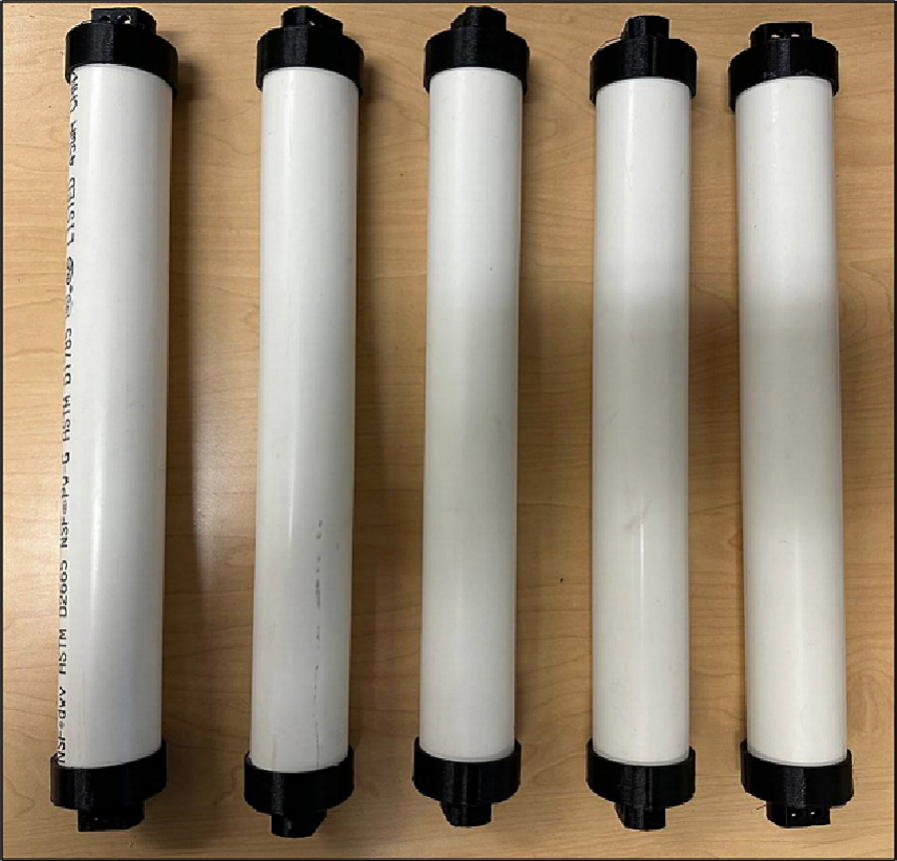
Gluing Roller Mounts on the PVC pipes.
4. Insert the ball bearings inside the Outer Plates holes (Fig. 19).

**Fig. 19:**
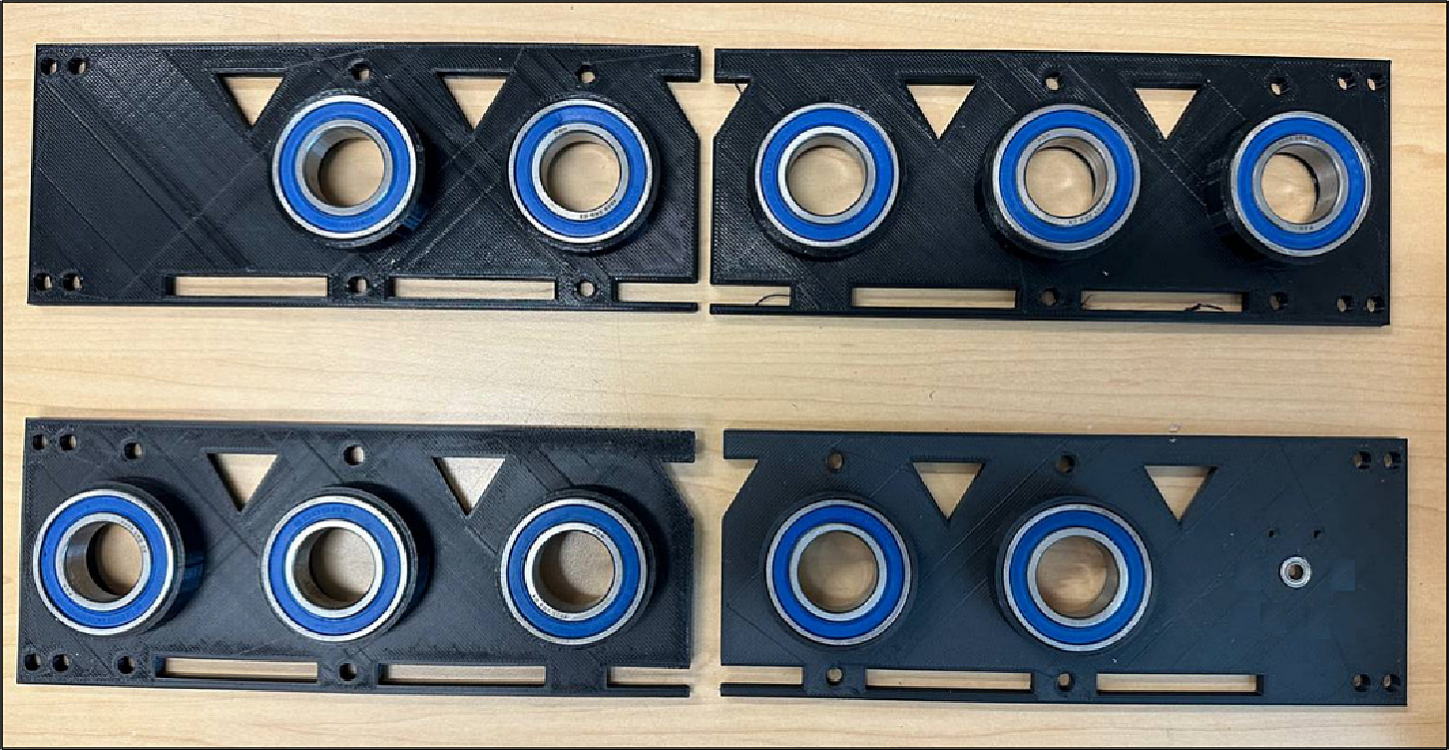
Inserting ball bearings inside the Outer Plates holes.
5. Bolt the half connectors on the Outer Plates (Fig. 20).

**Fig. 20:**
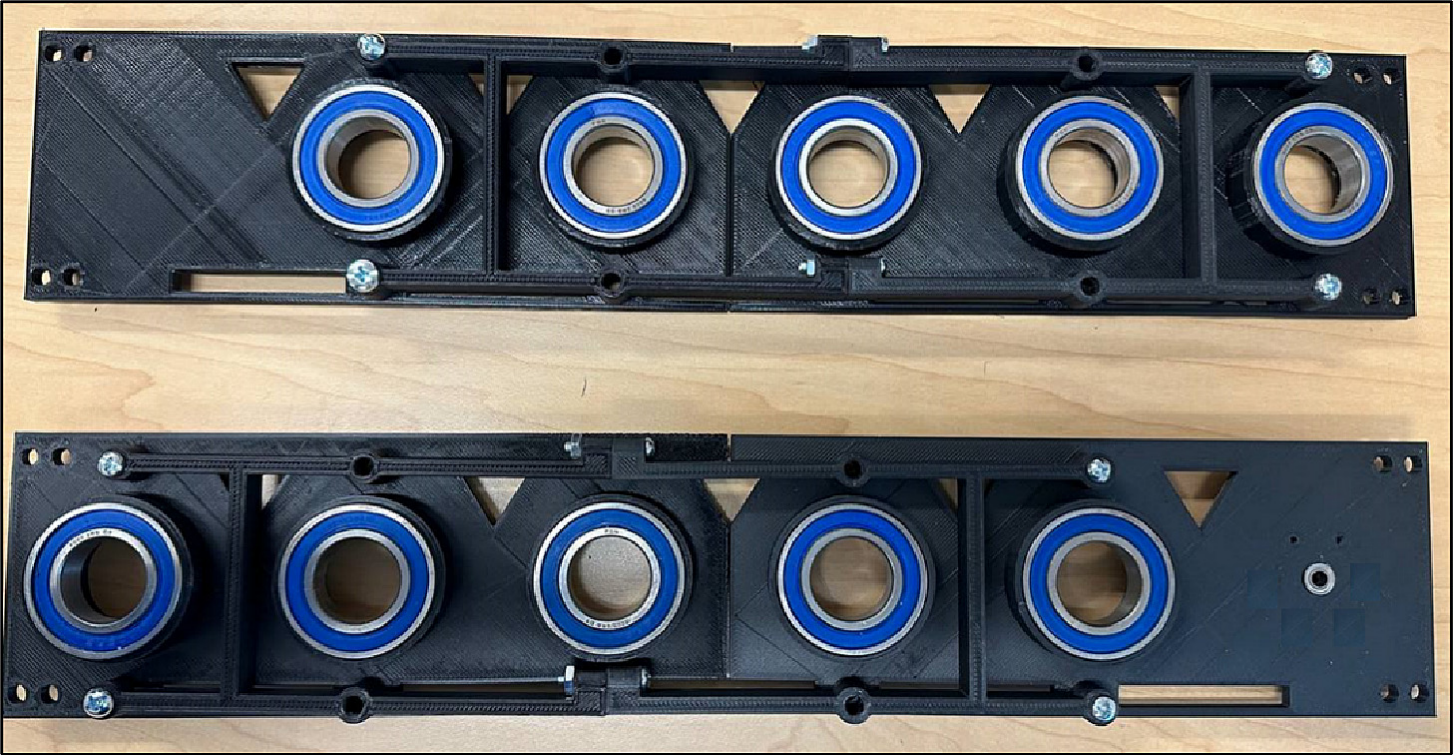
Bolting Half Connectors on the Outer Plates.
6. Bolt Reinforce Bars on the Half Connectors (Fig. 21).

**Fig. 21:**
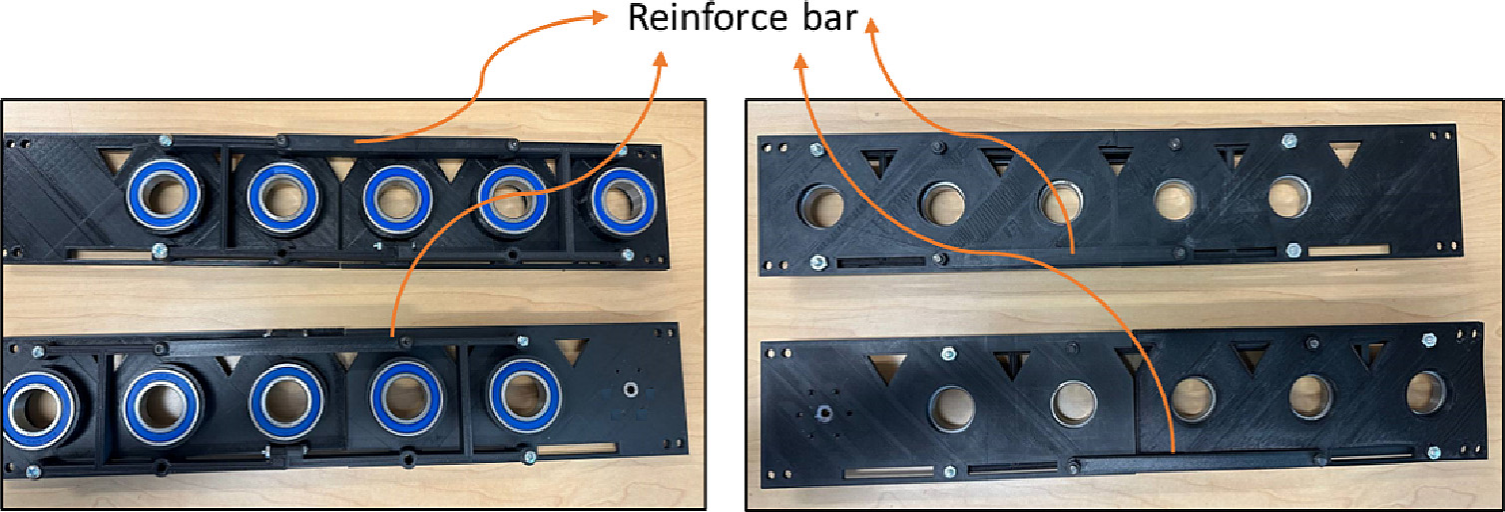
Screwing Reinforce Bars on Half Connectors.
7. Bolt walls together Fig. 22.

**Fig. 22:**
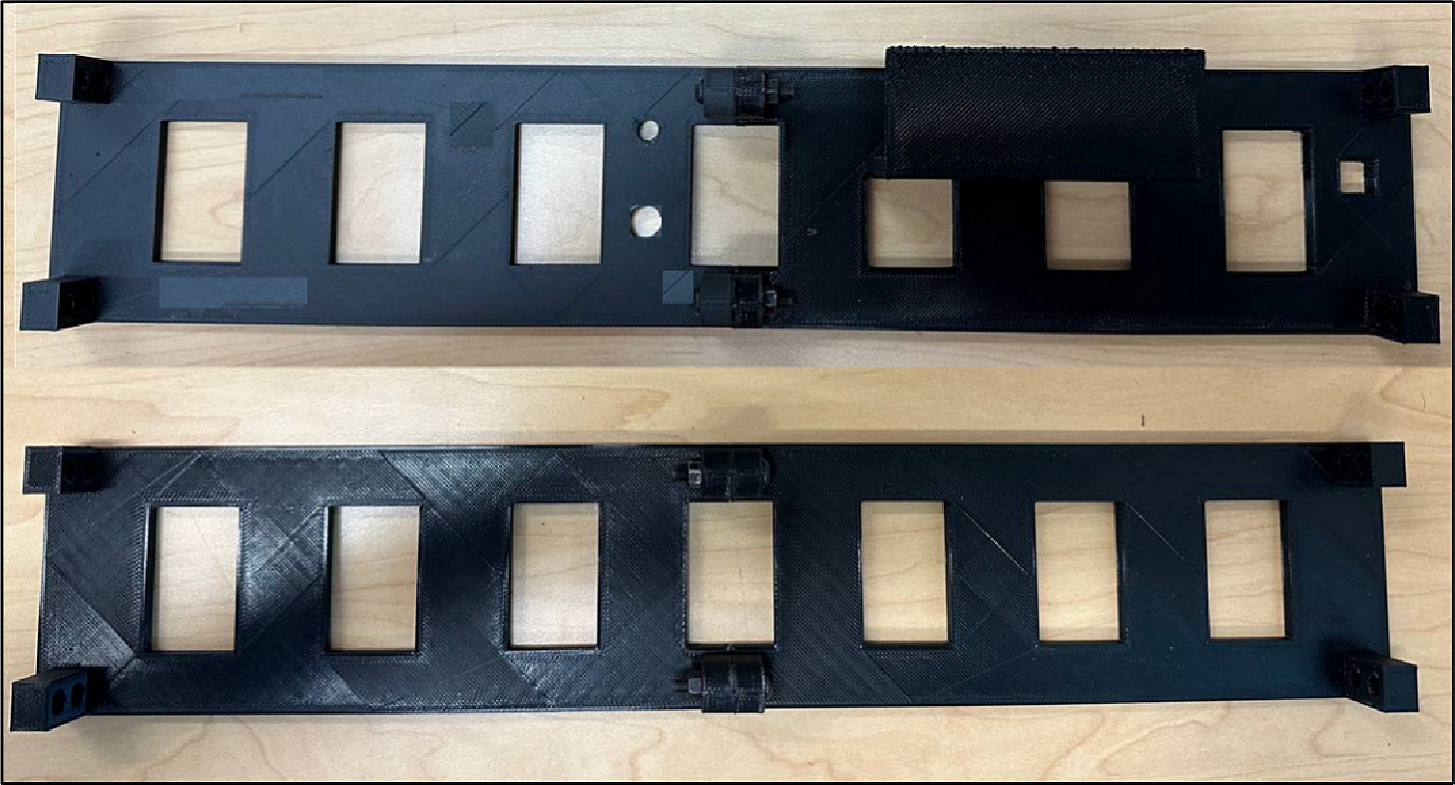
Bolting walls together.
8. Screw the walls to the Outer Plate-1 and Outer Plate-2 (Fig. 23).

**Fig. 23:**
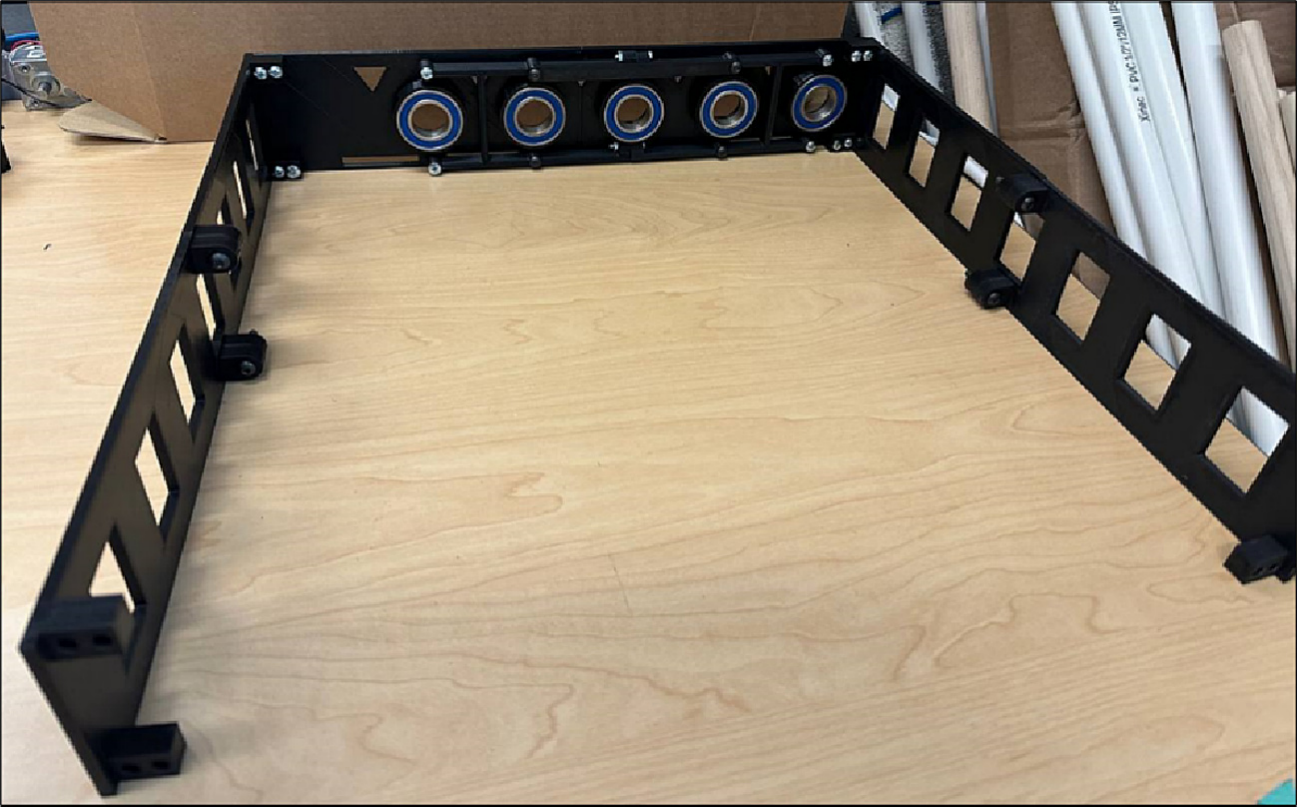
Screwing the walls to the Outer Plates.
9. Insert the Axis, Spacer, and PVC pipe in the ball bearings of Outer Plate-1 and Outer Plate-2 and repeat this step for 5 pipes based on Fig. 24.

**Fig. 24:**
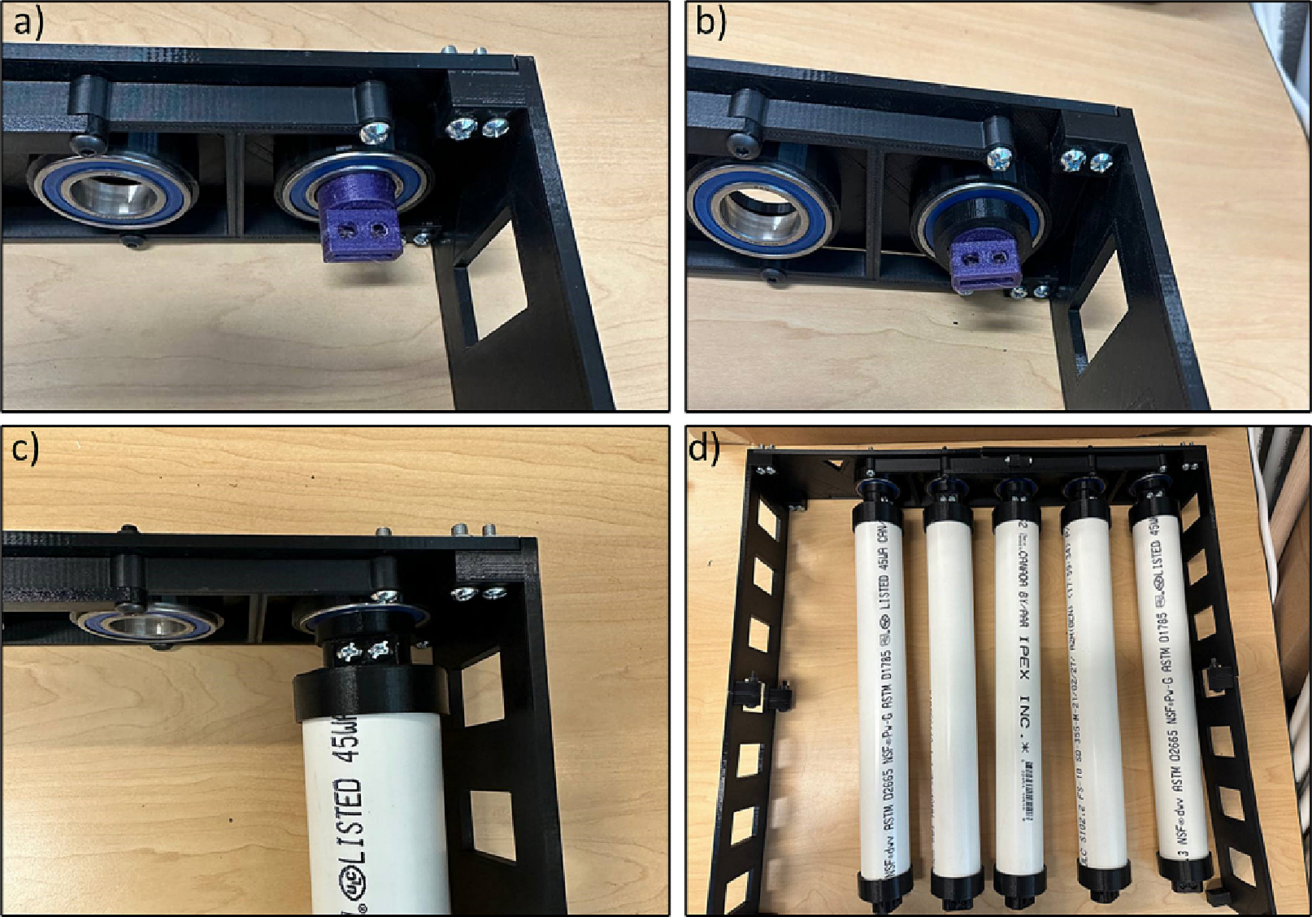
Inserting Axis, Spacer, and PVC pipe on the Outer Plate-1 and Outer Plate-2.
10. Screw the Outer Plate-3 and Outer Plate-4 on the walls (Fig. 25). Moreover, the key notes for the pipes assembly is shown in Fig. 26.

**Fig. 25:**
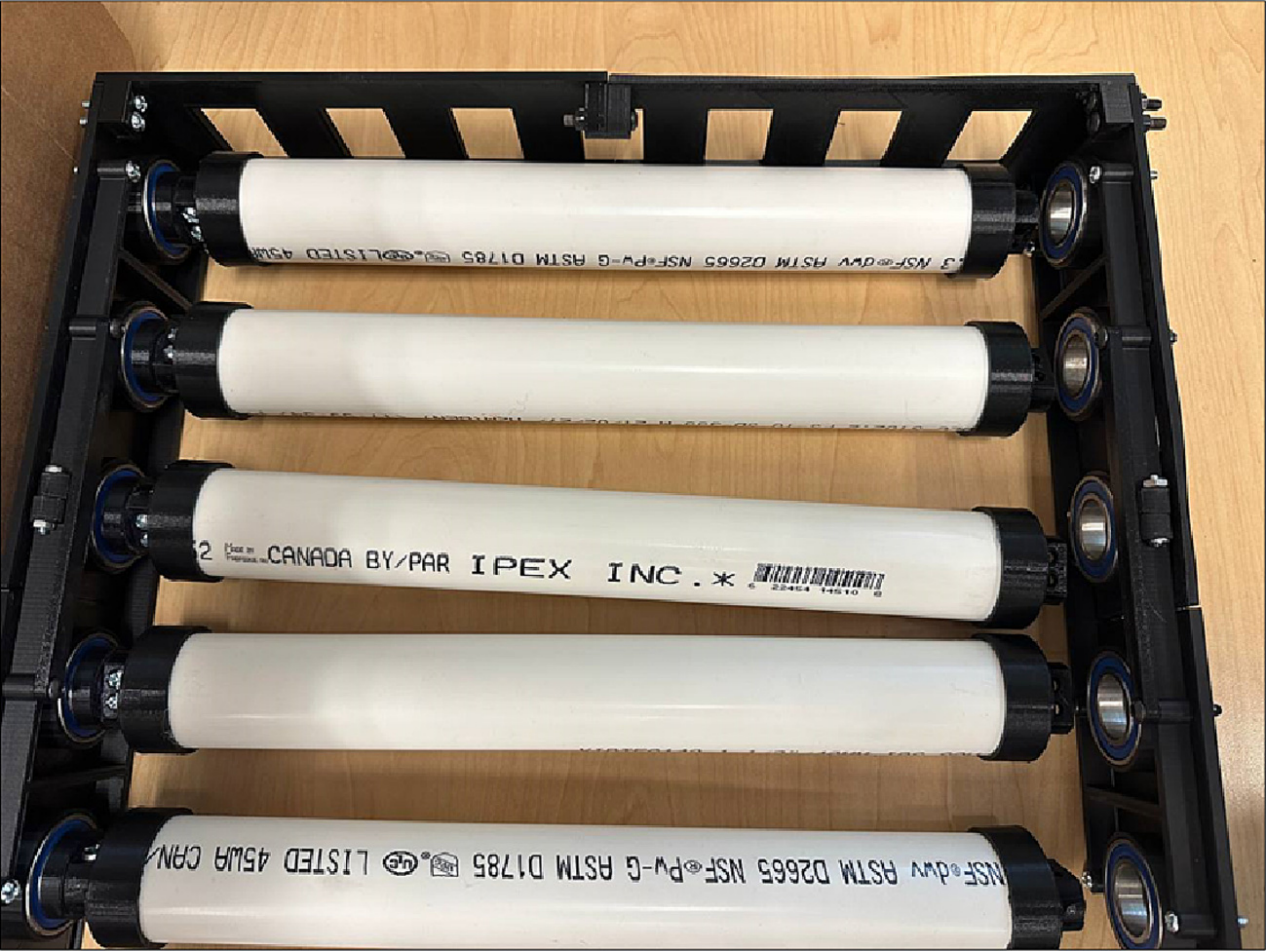
Screwing the Outer Plate-3 and Outer Plate-4 on the walls.

**Fig. 26:**
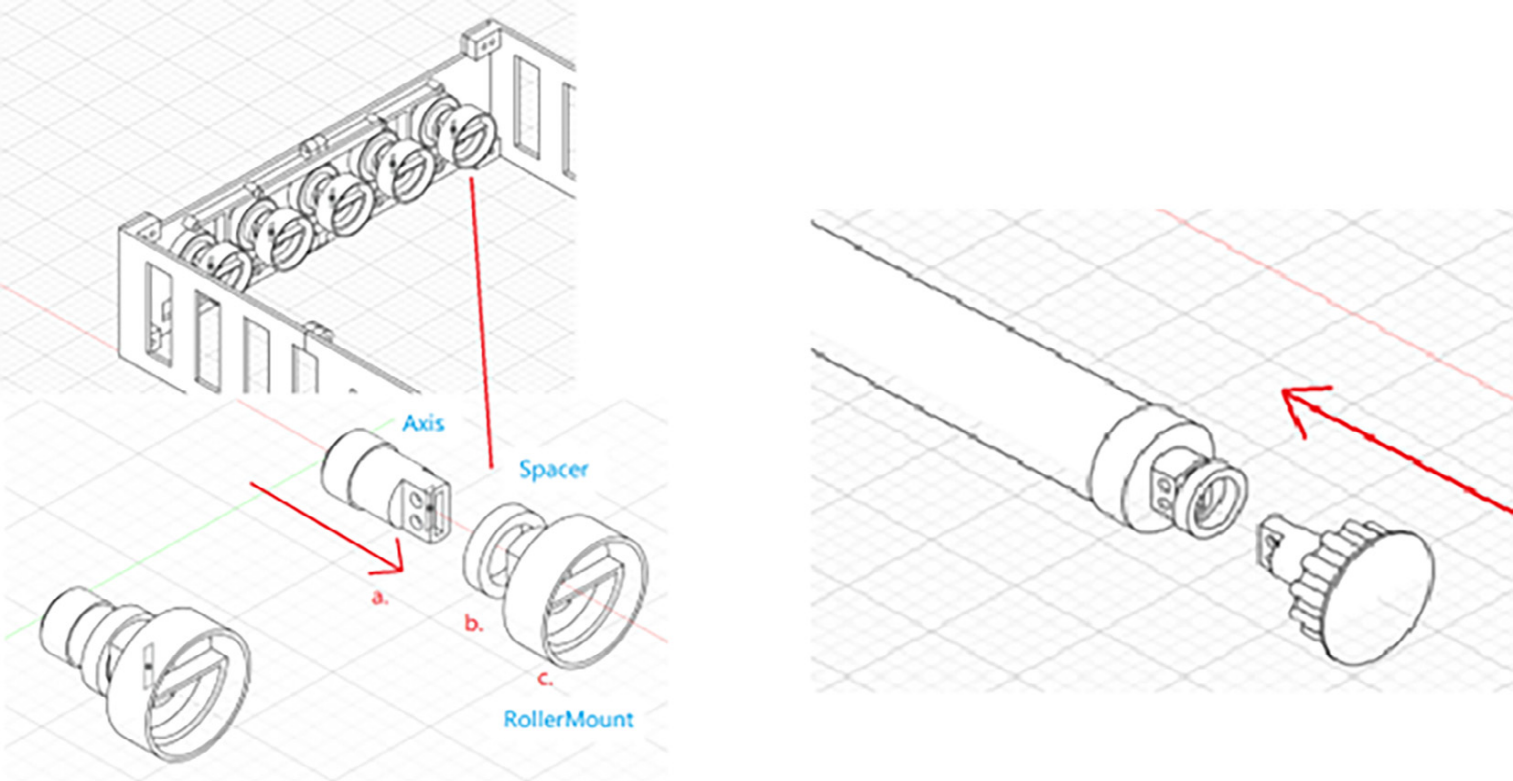
Pipes assembly.
11. Insert the Gear, the spacer, and PVC pipes in the ball bearings of Outer plate-3 and Outer plate-4 and repeat the step for all pipes with every other Gear (Gear-1 and Gear-2) (Fig. 27).

**Fig. 27:**
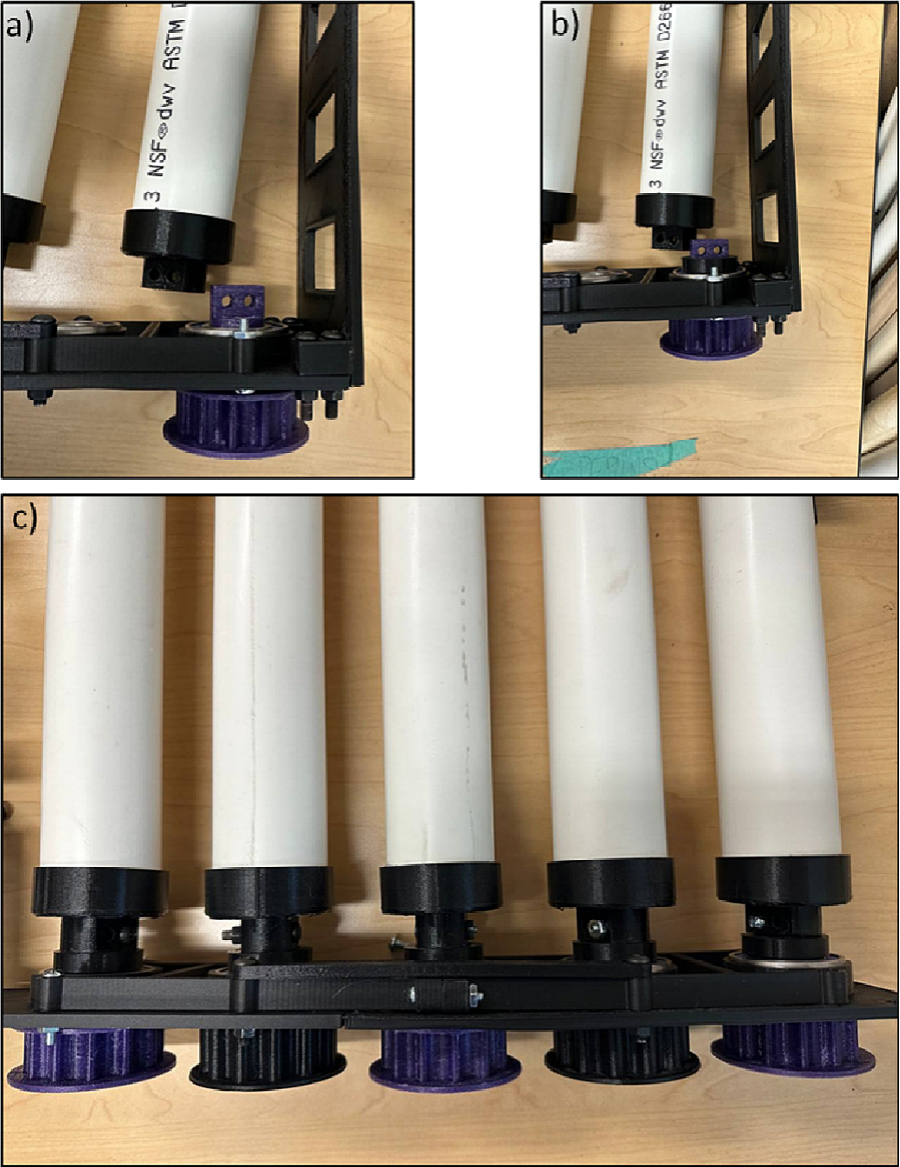
Inserting Gear-1 and Gear-2, the spacer, and the PVC pipes on Outer Plate-3 and Outer Plate-4.
12. Assemble the electronic components according to the electrical diagram (Fig. 28).

**Fig. 28:**
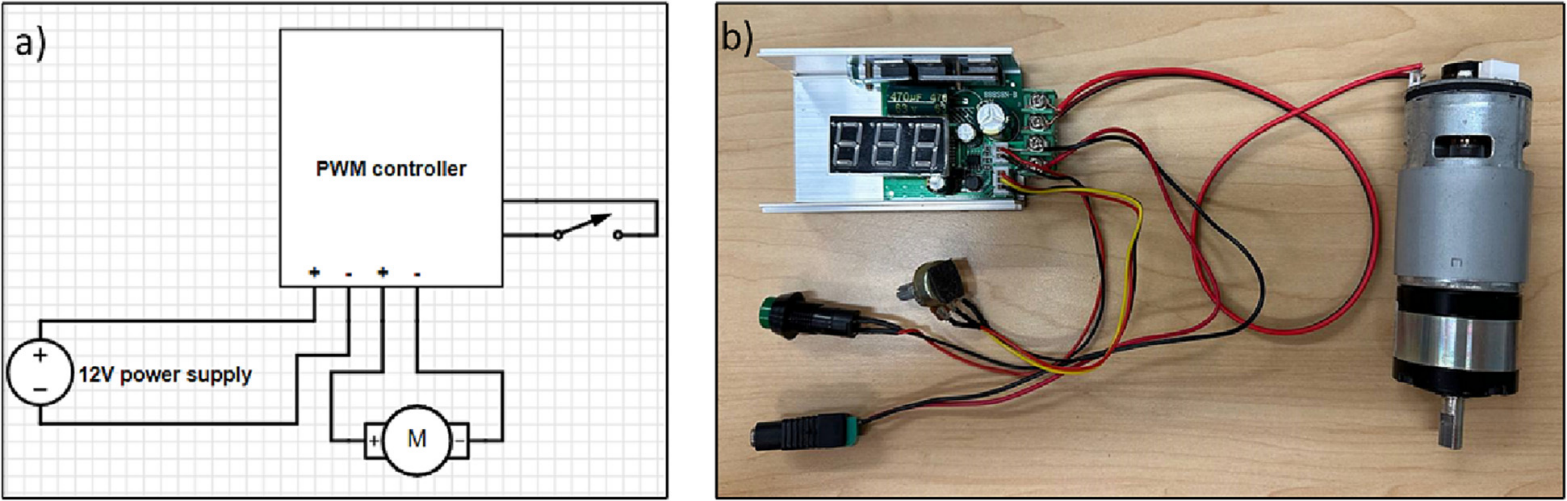
Assembling electronic components, a) Electrical diagram, b) assembles electronic parts.
13. Insert the motor into the Motor Box and attach them to the Outer Plate-3 (Fig. 29).

**Fig. 29:**
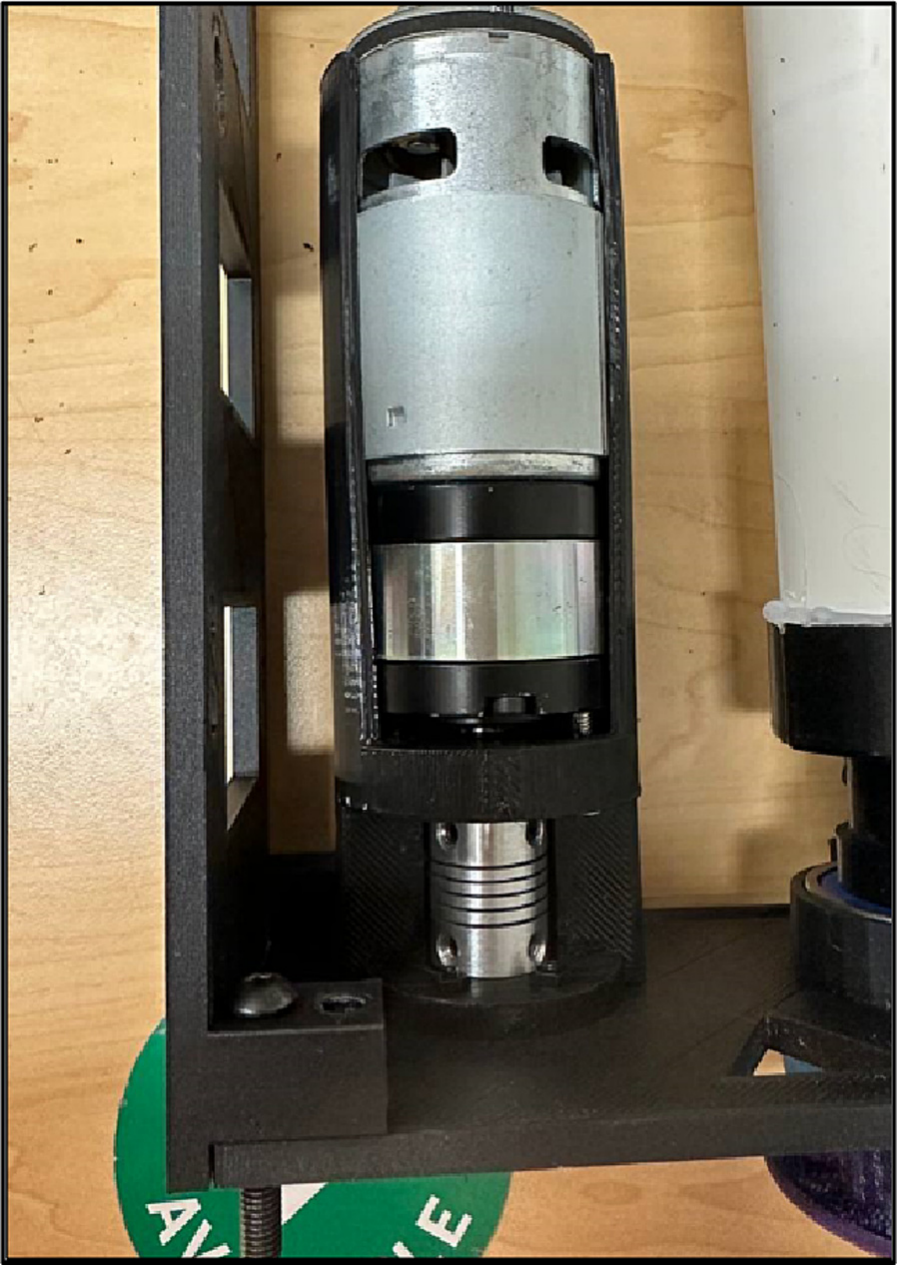
Inserting motor to the Motor Box and attaching them to the Outer Plate-3.
14. Insert the Belts as shown in Fig. 30.

**Fig. 30:**
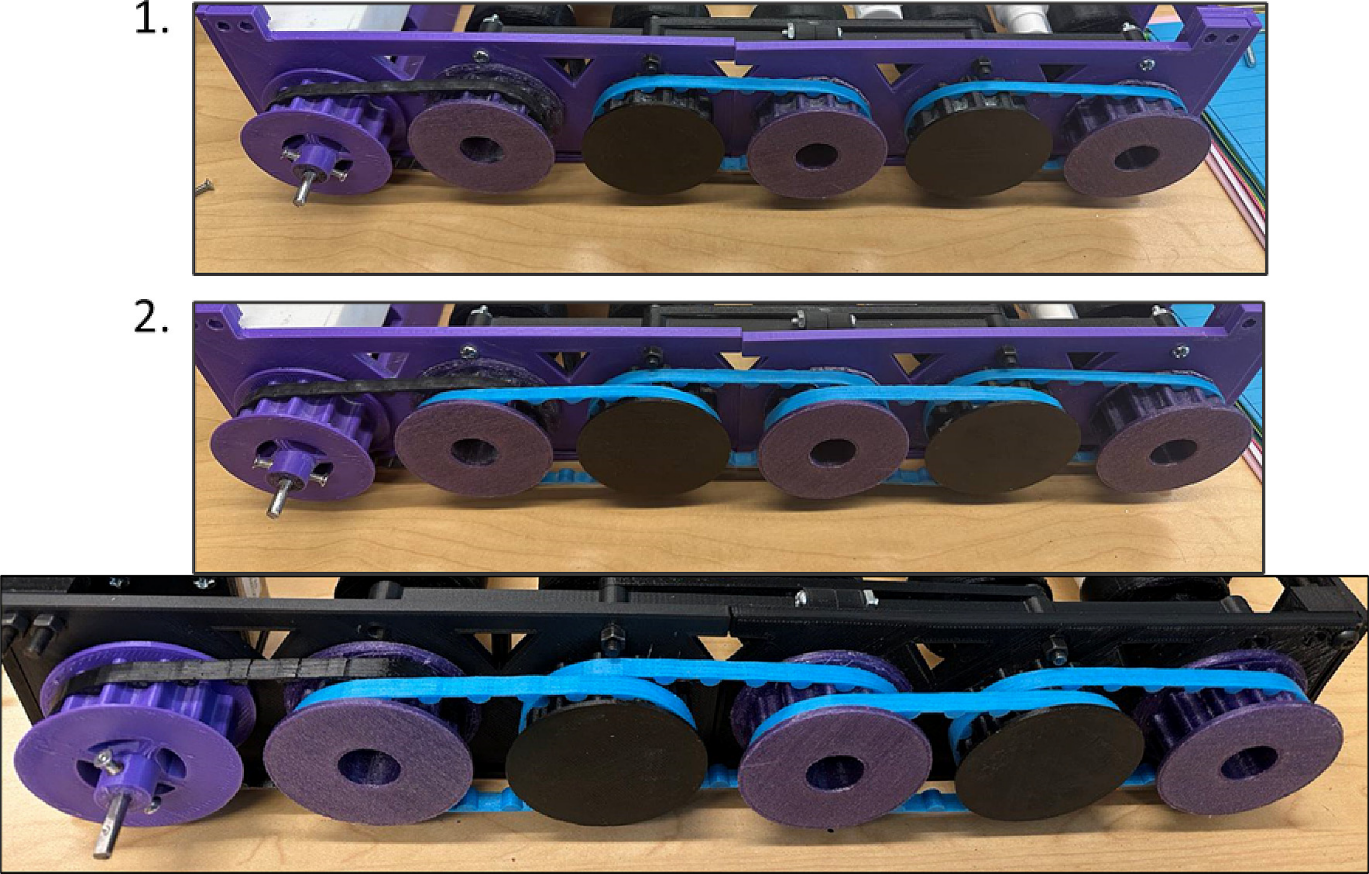
Adding the Belts.
15. Add the covers and electronic parts to the system and finish the assembling Fig. 31.

**Fig. 31:**
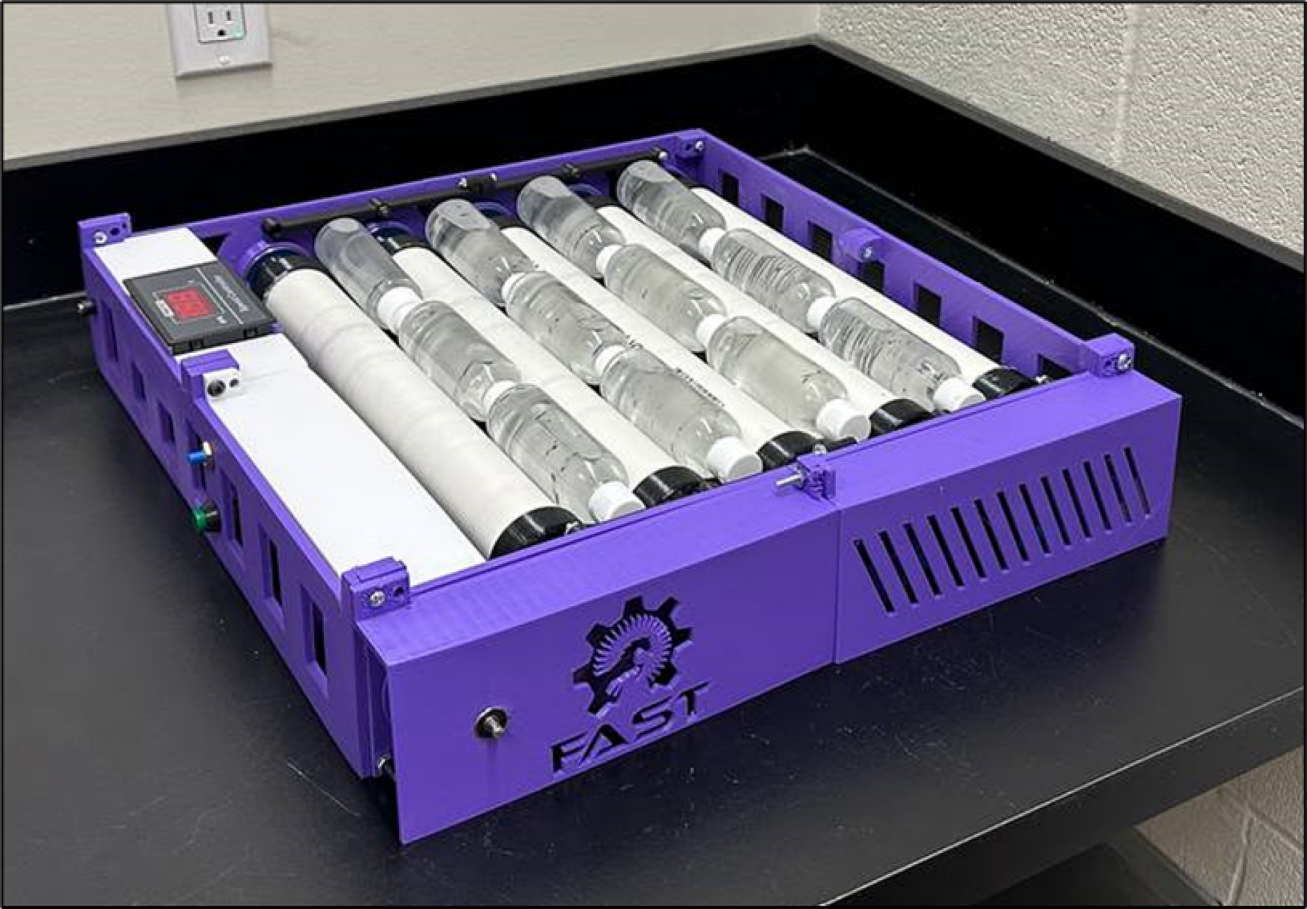
Adding covers and final assembly.

The operation instruction is as following:

1. Plug in the 12 V power supply.
2. Push the button to start the system.
3. Use the knob to adjust the power output to control the motor speed.
4. when the user wants to power it off or temporarily stop the operation, reverse the steps 1–3.

### Safety

There are several safety hazards that should be considered while working with the open source bottle roller. First, the bottle must be tightly sealed before putting on the bottle roller in case of the liquid spill onto the electronic part to cause a shortcut and potential electric shock. Secondly, the assembly of electronic components may require soldering, and users must follow the safety instructions during the soldering process to prevent injury. Furthermore, in order to prevent fingers from getting caught under the rollers, the operator should ensure that the speed is set to zero RPM and turn off the device using the speed controller.

## Validation and characterization

In this study, the performance of the open source bottle roller design was evaluated by comparing it to commercially available options. For this purpose, the information on the specifications and performance were collected as are provided in Table 1. The variables that will be investigated are: 1) the ability to maintain the appropriate speed that is required for the bottle roller to be effective over the duration of the desired amount of time at room temperature, and 2) the bottle roller ability to maintain constant speed while maintaining structural integrity in an oven at 50 °C.

Validation testing for the open-source bottle roller was designed to test the durability of the design in a heated environment for an extended period of time and be able to function at the same level as a commercial bottle roller. Validation testing took place for 48 h by placing twelve roller bottles completely filled with water, which had a weight of 100 mL per bottle. The speed controller allows fixing the speed at a precise number, including 80 rpm. With the open-source bottle roller fully loaded it was placed in a 50 °C oven with the rotation speed on the bottle roller set to 80 RPM. The bottle roller design and components were validated by maintaining full functionality after 24 h of continuous operation in the 50 °C oven. The operation of the open source bottle roller can be seen in two supplementary videos [52], [53].

The capabilities of the open source bottle roller include:

• The capital cost is reduced to CAD$210, which is 86% less expensive than the most affordable commercial bottle roller shown in Table 1.
• Capability of rolling 12 bottles filled with 100 mL water for at least 48 h without failure in room temperature at 80 RPM speed. Although the motor became slightly warm after several days of operation, it functioned safely without raising any concerns about causing injury during use or failure. Ability to operate for 24 h at elevated temperatures (50 °C) without failure.
• The bottles roll smoothly on the PVC pipes. To enhance the friction between the bottles and the pipes for applications with heavier masses, the pipes can be sanded or have rubber added to create a rougher/higher surface friction surface.
• Having a broader range of speeds offers the advantage of utilizing the bottle roller for other scientific applications. For instance, this device can be used to achieve homogeneous dispersion of silicon particles into polymer resin for 3-D printing of silicon-based objects via an SLA printer.
• Since the open-source bottle roller is customizable, it can accommodate different sizes of bottles by using PVC pipes with various dimensions.
• The device includes multiple belts that can be easily removed when the user needs to rotate fewer bottles. By removing belts, the user can reduce the number of pipes that roll, optimizing the device's performance for their specific application.

## Future work

The open source bottle roller was designed to be manufactured by most desktop 3-D printers. This asset, however, can be a limitation as because of the printer bed size being smaller than the device, the main body of the bottle roller is separated into eight pieces, which significantly impacts the structural strength. The connectors and reinforcement bars are designed to overcome this limitation by connecting these pieces together, but they result in the excess of printed parts, a slight increase in the height of the system and added costs for connectors. There are several open source approaches to solving this issue. First, a large format open source 3-D printer could be used to print the entire structural frame in a single print. If the large format printer was also a waste plastic fused granular fabrication (FGF)-based 3-D printer (cartesian [54], delta [55], or hang printer [56], [57]) the costs of the system could be reduced further by about 10% as recycled PETG particles could be used instead of filament. For example, the bottle roller could be fabricated by waste plastic PET water bottles [58]. Moreover, by selecting a higher melting point 3-D printable polymer like polycarbonate (PC) even higher temperature operation may be possible [59]. Another potential solution to this design limitation is to utilize open-source laser cutting or CNC milling [60] to cut the four sides of the bottle roller on a plastic sheet. Further the plastic sheets could be fabricated in an open source hot press from recycled plastic as well [61]. This way, the bottle roller can be held in place and the connectors and reinforcement bars can be eliminated and ensure overall strength and stability. In addition, to reduce the purchased components the PVC pipes can be replaced by 3-D printed pipes or extrusion molded pipes from an open source recyclebot [62], [63], [64], [65], [66].

**Fig. 32:**
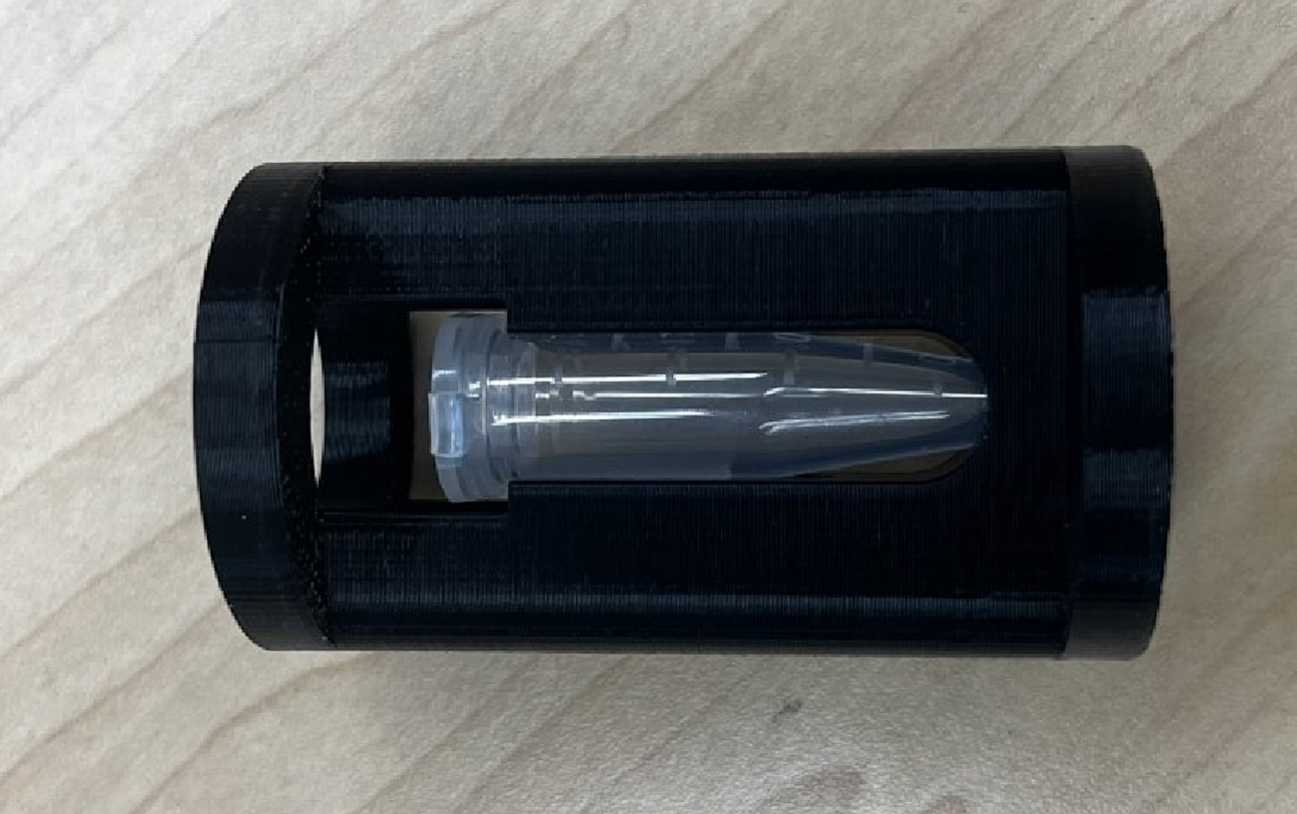
3-D printable adapter for microcentrifuge tube.

Moreover, the bottle roller is customizable to be used in different scale applications. The smaller version can be obtained using PVC pipes with smaller diameter. It should be pointed out that the same percentile cost savings could be had by using either filament from an open-source recyclebot or small-scale FGF [67], [68]. In this regard, changing the dimensions of the provided designs can be helpful. Also, the user can make the bigger version through adding outer plates. The other design that can be useful and adapted easily from the current design for some applications is the stacked version that consist of some bottle rollers on top of each other. This would be helpful for scaling bottle rolling applications to greater production volumes. The bottle roller can also be used for non-cylindrical components or specialty vessels. To do this a 3-D printed component can be fashioned to hold these non-optimal vessels. To illustrate this Fig. 32 shows a 3-D printable holder for a microcentrifuge tube, which can then be used on the open source bottle roller. Similar strategies can be used for many microcentrifuge tubes or other types or shapes of containers.

## Ethics statements

This project does not involve human subjects and animal experiments.

## CRediT authorship contribution statement

**Maryam Mottaghi:** Methodology, Validation, Formal analysis, Investigation, Data curation, Writing – original draft, Writing – review & editing, Visualization. **Yuntian Bai:** Methodology, Validation, Formal analysis, Writing – review & editing, Visualization. **Apoorv Kulkarni:** Methodology, Software, Validation, Formal analysis, Writing – review & editing, Visualization. **Joshua M. Pearce:** Conceptualization, Methodology, Formal analysis, Resources, Data curation, Writing – original draft, Writing – review & editing, Supervision, Funding acquisition.

## Declaration of Competing Interest

The authors declare that they have no known competing financial interests or personal relationships that could have appeared to influence the work reported in this paper.
